# Gut microbiota-dependent increase in phenylacetic acid induces endothelial cell senescence during aging

**DOI:** 10.1038/s43587-025-00864-8

**Published:** 2025-05-12

**Authors:** Seyed Soheil Saeedi Saravi, Benoit Pugin, Florentin Constancias, Khatereh Shabanian, Marianne Spalinger, Aurélien Thomas, Sylvain Le Gludic, Taraneh Shabanian, Gergely Karsai, Manuel Colucci, Cristina Menni, Ilias Attaye, Xinyuan Zhang, Meret Sarah Allemann, Pratintip Lee, Alessia Visconti, Mario Falchi, Andrea Alimonti, Frank Ruschitzka, Francesco Paneni, Jürg H. Beer

**Affiliations:** 1 Center for Translational and Experimental Cardiology, Department of Cardiology, University Hospital Zurich, University of Zurich, Schlieren, Switzerland; 2 University Heart Center, Department of Cardiology, University Hospital Zurich, Zurich, Switzerland; 3 Laboratory of Food Biotechnology, Institute of Food, Nutrition and Health, Department of Health Sciences and Technology, ETH Zurich, Zurich, Switzerland; 4 Department for Gastroenterology and Hepatology, University Hospital Zurich, University of Zurich, Zurich, Switzerland; 5 Faculty Unit of Toxicology, University Center of Legal Medicine, Lausanne University Hospital and University of Lausanne, Lausanne, Switzerland; 6 Unit of Forensic Toxicology and Chemistry, University Center of Legal Medicine, Lausanne University Hospital and University of Lausanne, Geneva University Hospital and University of Geneva, Lausanne, Geneva, Switzerland; 7 Institute of Clinical Chemistry, University Hospital Zurich, Schlieren, Switzerland; 8 Institute of Oncology Research (IOR), Bellinzona, Switzerland; 9 Università della Svizzera Italiana, Lugano, Switzerland; 10 Department of Twin Research, King’s College London, St Thomas’ Hospital Campus, London, UK; 11 Department of Pathophysiology and Transplantation, Università Degli Studi di Milano, Milan, Italy; 12 Fondazione IRCCS Cà Granda Ospedale Maggiore Policlinico, Angelo Bianchi Bonomi Hemophilia and Thrombosis Center, Milan, Italy; 13 Amsterdam Cardiovascular Sciences, Diabetes & Metabolism, Amsterdam, Netherlands; 14 Center for Molecular Cardiology, University of Zurich, Schlieren, Switzerland; 15 Department of Internal Medicine, Cantonal Hospital Baden, Baden, Switzerland; 16 Centre for Biostatistics, Epidemiology, and Public Health, Department of Clinial and Biological Sciences, University of Turin, Turin, Italy; 17 Department of Medicine, University of Padova, Padova, Italy; 18 Department of Health Sciences and Technology (D-HEST), ETH Zurich, Zurich, Switzerland; 19 Oncology Institute of Southern Switzerland, Ente Ospedaliero Cantonale, Bellinzona, Switzerland

**Keywords:** Senescence, Microbiology, Ageing

## Abstract

Endothelial cell senescence is a key driver of cardiovascular aging, yet little is known about the mechanisms by which it is induced in vivo. Here we show that the gut bacterial metabolite phenylacetic acid (PAA) and its byproduct, phenylacetylglutamine (PAGln), are elevated in aged humans and mice. Metagenomic analyses reveal an age-related increase in PAA-producing microbial pathways, positively linked to the bacterium *Clostridium* sp. ASF356 (*Clos*). We demonstrate that colonization of young mice with *Clos* increases blood PAA levels and induces endothelial senescence and angiogenic incompetence. Mechanistically, we find that PAA triggers senescence through mitochondrial H_2_O_2_ production, exacerbating the senescence-associated secretory phenotype. By contrast, we demonstrate that fecal acetate levels are reduced with age, compromising its function as a Sirt1-dependent senomorphic, regulating proinflammatory secretion and redox homeostasis. These findings define PAA as a mediator of gut–vascular crosstalk in aging and identify sodium acetate as a potential microbiome-based senotherapy to promote healthy aging.

## Abstract (web summary)

Saeedi Saravi and colleagues demonstrate an age-related increase in the gut microbial metabolite phenylacetic acid (PAA) in humans and mice, linked to *Clostridium* bacteria. In mice, they find that PAA triggers endothelial senescence, linking the microbiome to vascular aging.

## Main

Aging is a substantial risk factor for prevalent cardiovascular diseases (CVDs), affecting millions of people worldwide^1^. The alarming rise in CVD incidence in older adults emphasizes our inadequate understanding of its development with age^2^. Dysfunctional endothelial cells (ECs) are increasingly recognized to contribute to CVDs^3^. Advanced age causes cellular senescence in ECs, a crucial factor in EC dysfunction in older adults^4^. Senescent ECs (SECs) undergo a variety of persistent alterations, including epigenetic modifications, irreversible cell-cycle arrest, DNA instability and the senescence-associated secretory phenotype (SASP), which impairs angiogenic capacity and vascular function^5^. Therefore, accumulation of SECs is considered a primary driver to aging-related CVDs.

Oxidative stress is one identified trigger of stress-induced senescence in ECs^6^. Converging evidence suggests that gut microbiota, a yet underappreciated endocrine organ, regulates oxidative-regulated host genes involved in endothelial biology^7^. Although such studies have linked gut microbiota with CVD^8^, there is limited evidence on its role in EC senescence and the molecular mechanisms in aging organisms.

With advanced age, alterations of specific gut-derived metabolites such as aromatic amino acid derivatives might contribute to CVD through prolonged oxidative stress and inflammation^9^. One such example is phenylacetic acid (PAA) and its derivative phenylacetylglutamine (PAGln), which are produced from phenylalanine by commensal microbial *porA* or *ppfor* genes^10^. These metabolites are positively associated with cardiovascular and all-cause mortality in patients with chronic kidney disease (CKD)^11^ and heart failure^12^. In preclinical models, PAA stimulated reactive oxygen species (ROS) production and further inflammation in ECs^13^, explaining its role in vascular damage in CKD. Similarly, PAA-derived PAGln fostered ROS production and apoptosis in cardiomyocytes, compromising their contractility^12^. In this study, we observed higher plasma concentrations of PAA and PAGln in both the TwinsUK human aging cohort and aged mice, hypothesizing that elevated levels of these two metabolites during aging may actively promote EC senescence and vascular dysfunction. We aimed to uncover mechanisms linking aging gut-derived PAA with EC senescence and dysfunction and to explore microbiome-based therapeutic strategies for senescence rescue and restoring angiogenic capacity in these cells.

Beyond the metabolites, gut microbiota produces a myriad of other metabolites like short-chain fatty acids (SCFAs), mainly including acetate, propionate and butyrate, which circumvent atherosclerosis and other CVD risk factors^9^. It is well characterized that >70% of colonic acetate, but only small amounts of propionate and butyrate, reach circulation after hepatic metabolism^14^. We have previously reported that aging markedly reduces fecal acetate levels by up to 80% due to the depletion of acetate-producing bacteria, such as *Prevotella* and *Rikenellaceae*_RC9_gut_group^15^. Notably, the depletion of these bacteria in the microbiome has been ubiquitously observed in CVD and is associated with disease severity^16^. Converting acetate to acetyl-CoA in mitochondria, quiescent ECs can sustain the tricarboxylic acid (TCA) cycle and redox homeostasis, protecting themselves against oxidative stress^17^. Acetate also controls epigenetic alterations by regulating histone acetylation/deacetylation critical to EC phenotype and function^17^. Accordingly, we hypothesized that exogenous acetate might mitigate PAA-induced oxidative stress and associated epigenetic alterations driving EC senescence, offering therapeutic potential for EC dysfunction in aging. Notably, our findings suggest acetate may help ECs escape senescence and protect against age-related arterial dysfunction and associated CVD.

## Results

### Gut microbiota link to elevated PAA and PAGln in aging

**Fig. 1:**
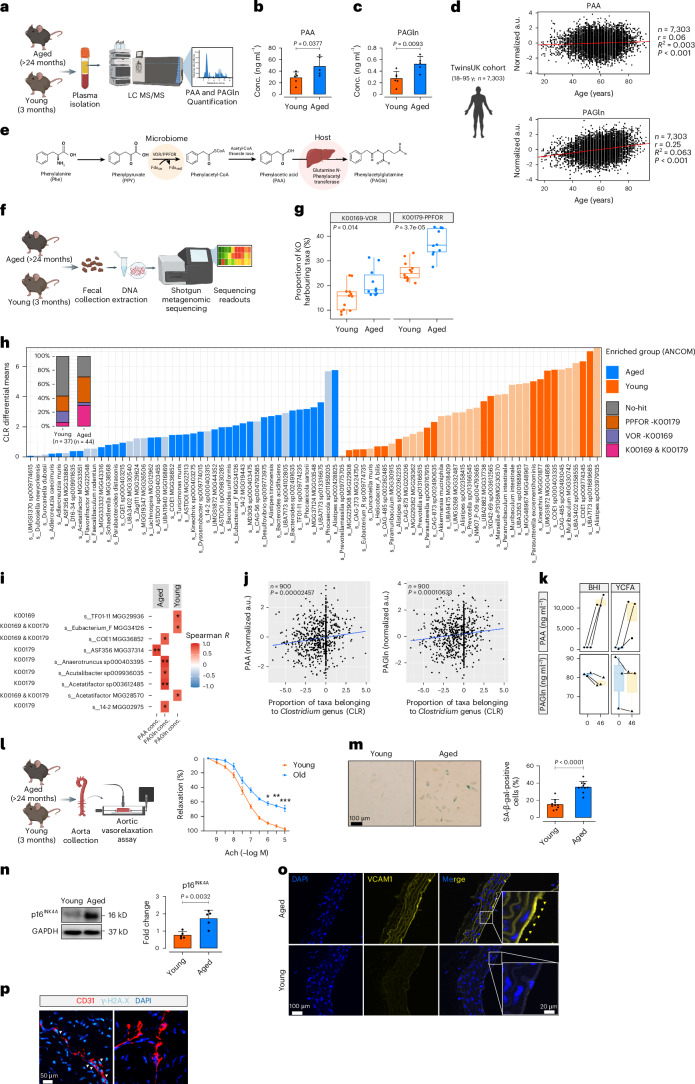
Reflection of age in plasma levels of gut microbiota-derived metabolites PAA and PAGln. **a**, Schematic of quantification of plasma PAA and PAGln in young (3-month-old) and aged (>24-month-old) C57BL/6J male and female mice. **b**,**c**, Plasma PAA (**b**) and PAGln (**c**) levels were measured by LC–MS/MS in mice (*n* = 6). **d**, Correlation between plasma PAA (left) and PAGln (right) concentrations and chronological age in the TwinsUK cohort (*n* = 7,303, male and female). **e**, Schema for PAA production from dietary phenylalanine (Phe) via the bacterial VOR/PPFOR system. **f**, Shotgun metagenomics workflow for analyzing mouse fecal microbiomes. **g**, Distribution of VOR and PPFOR gene homologs (%) in microbiomes of aged and young mice (*n* = 5–6). KOO169, VOR; KOO179, PPFOR). **h**, Bar plot depicting age-associated abundance of fecal microbiota profiles at strain level (dark colors represent taxa harboring VOR or PPFOR homologs). The leftmost bar plot demonstrates the proportion of VOR, PPFOR or VOR + PPFOR detected in taxa enriched in aged (*n* = 44) versus young (*n* = 37) mice. **i**, Heatmap shows correlations between plasma PAA or PAGln levels and gut bacteria among the top enriched taxa in aged versus young mice (*n* = 5–6). **j**, Correlation between plasma PAA (left) and PAGln (right) concentrations and abundance (%) of *Clostridium* taxa in human participants (*n* = 900, TwinsUK; male and female). **k**, PAA (top) and PAGln (below) concentrations in the supernatants of anaerobic cultures from *Clostridium* sp. ASF356 (*n* = 8). **l**, Schematic of ex vivo force tension myography. Aortic rings exhibit vasorelaxation responses (%) to acetylcholine (Ach) (*n* = 10). **m**, SA-β-gal staining of ECs from the ascending aortas of mice (*n* = 5–6) and quantification of SA-β-gal^+^ cells (%). **n**,**o**, Immunoblots and immunofluorescence represent the expression of p16^INK4a^ (**n**) and VCAM1 (**o**) in aortic ECs (*n* = 5–6). **p**, γ-H2A.X immunostaining in CD31^+^ ECs (*n* = 5–6). Scale bars, 20, 50 and 100 μm. Error bars represent s.d. (**b**,**c**,**m**,**n**) or s.e.m. (**g**,**k**,**l**) or 95% confidence intervals (**d**). Statistical analysis was performed using a two-tailed unpaired Student’s *t*-test (**b**,**c**,**l**–**n**), two-tailed Pearson correlation analysis (**d**), two-tailed Mann–Whitney *U*-test (**g**,**k**), ANCOM method for microbial abundance analysis (**h**), two-sided Spearman’s rank correlation test (**i**) and linear mixed model CLR transformation (**j**). Data are shown as median with min–max; boxes represent interquartile range (IQR); center lines represent the median; whiskers extend from the min to max values (**g**,**k**). Images created with BioRender.com (**a**,**d**–**f**,**l**). *P < 0.05, **P < 0.01, ***P < 0.001. Source data

Our data identified markedly higher plasma concentrations of PAA and PAGln in aged mice (>24 months) compared to young mice (>3 months), as detected by targeted metabolomics (Fig. 1a–c). To rule out kidney function effects on age-related increases in plasma levels of PAA and PAGln, mice were matched for sex, body weight and renal function (Supplementary Fig. 1a–c). Similar trends were observed in data from the TwinsUK human cohort (https://twinsuk.ac.uk/), where nontargeted metabolomics confirmed age-associated increases in PAA and PAGln (Fig. 1d). Accordingly, linear regression analysis between metabolite concentrations and age revealed that both metabolites were age-dependently elevated in 7,303 individuals between 18–95 years old (PAA*, r* = 0.06, *R*^*2*^ = 0.003, *P* < 0.001; PAGln, *r* = 0.25, *R*^*2*^ = 0.063, *P* < 0.001).

**Extended Data Fig. 1:**
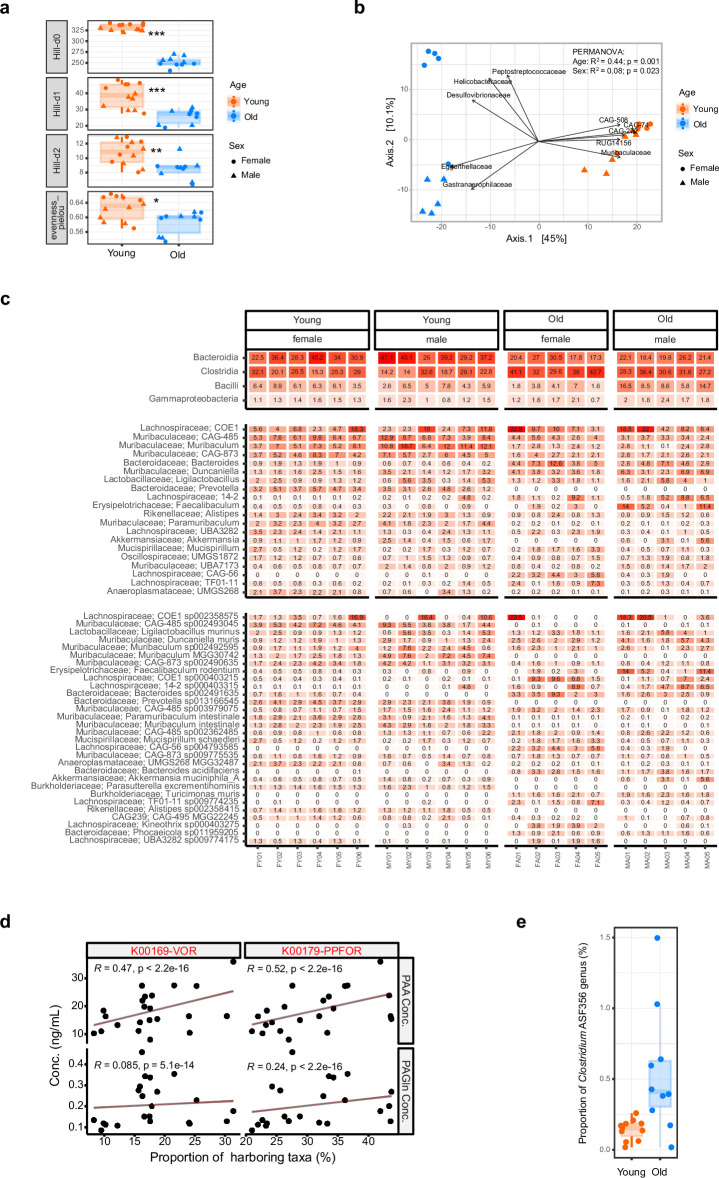
Age-associated gut microbiota alterations. **a**, Box plots representing alpha diversity in feces of female and male, old and young C57BL/6J mice measured by Hill-d0 to 2 and Pielou’s evenness indices (n = 5–6). **b**, Principal coordinate analysis (PCoA) plot showing beta diversity based on the community level changes in old and young mice (n = 5–6). **c**, Heatmap depicting differentially abundant microbial profiles at the strain level (each color represents one bacterial taxa) in the fecal microbiota of female and male, old *versus* young mice (n = 5–6). **d**, Gene–metabolite interactions explain a positive correlation of the proportion (%) of either VOR- or PPFOR-harboring taxa and plasma PAA levels in aged mice. Nevertheless, the lowermost chart shows non-significant association of VOR or PPFOR homologs and plasma PAGln levels in these mice (n = 5–6). **e**, Distribution of the *Clostridium* genus in mouse microbiomes. The box plots show the higher proportion (%) of taxa belonging to the *Clostridium* genus in the microbiome of aged mice compared to young mice (n = 5–6). Error bars represent SEM (**a, b, d, e**). Data were analyzed using Kruskal–Wallis statistical test (**a**), Atchinson distance and permutational multivariate analysis of variance (PERMANOVA) (**b**), partial Spearman’s correlation test tests (**d**), and two-tailed unpaired Student’s *t*-test (**e**). (*P < 0.05, **P < 0.01, ***P < 0.001). Source data are provided as a Source Data file. Source data

Previous studies have shown that gut microbiota convert dietary phenylalanine into PAA via a metaorganismal pathway, at which point host PAA-to-glutamine conjugation leads to PAGln generation^10^. Our supplementary and others^10,18^ studies revealed that antibiotic suppression of gut microbiota markedly reduces circulating PAA and PAGln levels, reinforcing gut microbial involvement (Supplementary Fig. 2a,b). Two distinct microbial pathways (phenylpyruvate:ferredoxin oxidoreductase (PPFOR) and α-ketoisovalerate:ferredoxin oxidoreductase (VOR)) contribute to PAA biosynthesis via oxidative decarboxylation of phenylalanine^10^ (Fig. 1e). To investigate whether the pathways dominate in aging, we performed fecal shotgun metagenomics within our mouse model of chronological aging (Fig. 1f). At the initial step, our data unsurprisingly revealed significant alterations in both α-diversity (richness and evenness) and β-diversity indices (Extended Data Fig. 1a,b) in the gut microbiome of aged mice. The Kyoto Encyclopedia of Genes and Genomes (KEGG) analysis identified a significantly higher abundance of *ppfor* and *vor* gene homologs in the microbiome of aged mice (*P* = 3.7 × 10^−5^ for *ppfor*; *P* = 0.014 for *vor*; Fig. 1g). A significant correlation between plasma PAA levels and percentage of either *ppfor*- or *vor*-positive genomes was also observed in the aged microbiomes (*R*^*2*^ = 0.52 for *ppfor* and *R*^*2*^ = 0.47 for *vor*, *P* < 0.0001; Extended Data Fig. 1d); however, plasma PAGln concentrations displayed weaker associations with *ppfor*- or *vor*-harboring bacteria in aged mice (*R*^*2*^ = 0.24 for *ppfor*; *R*^*2*^ = 0.085 for *vor*; Extended Data Fig. 1d). In line with the above findings, the centered log ratio (CLR) by analysis of composition of microbiomes (ANCOM) demonstrates differentially abundant bacterial taxa harboring VOR, PPFOR or both homologs in the aged microbiomes. Accordingly, a ~1.7-fold higher proportion of PPFOR or VOR + PPFOR homologs was enriched in aged mice compared to those in young mice (72% versus 42%; Fig. 1h). Focusing on specific bacteria linked to age-related PAA elevation, Spearman’s rank correlation analysis pinpointed *Clostridium* sp. ASF356 MGG37314 as the only PPFOR-positive bacterium positively associated with plasma PAA levels in aged mice (Fig. 1i). This bacterium was moderately abundant in aged microbiomes (ranging from 0.012099% to 1.400074%; Extended Data Fig. 1e). Five other taxa from the class *Clostridia* displayed a positive association with plasma PAGln, but not PAA, in aged mice. To translate our observations to humans, we conducted a taxonomic profiling in individuals enrolled in the TwinsUK study (*n* = 900). Our findings revealed a substantial enrichment of bacterial taxa belonging to the *Clostridium* genus (harboring *ppfor* gene) in older individuals, correlating more strongly with PAA (*P* = 0.00002457) than PAGln (*P* = 0.00010633) (Fig. 1j). To further validate the functional role of *Clostridium* sp. ASF356 MGG37314 in PAA formation, we cultivated an axenic culture and tested for PAA production in phenylalanine-containing brain heart infusion or yeast casitone fatty acids medium under anaerobic condition by liquid chromatography tandem mass spectrometry (LC–MS/MS) (Fig. 1k). These results suggest that *Clostridium* sp. ASF356 commensal in the aged microbiome contributes to higher circulating PAA levels.

Aged mice exhibited marked endothelial dysfunction, evidenced by reduced endothelium-dependent relaxation to acetylcholine. As shown in Fig. 1l, maximal relaxation at 10^−5^ M acetylcholine was significantly lower in aged mice (97.57 ± 1.91% in young versus 69.13 ± 4.25% in aged; *P* < 0.001) with a rightward dose-response shift (half maximal effective concentration (EC_50_) 34.23 ± 9.01 nM in young versus 51.33 ± 12.32 nM in aged; *P* < 0.05). This dysfunction coincided with a significant rise in cellular senescence hallmarks, including increases in lysosomal alteration, as indicated by SA-β-gal positivity (Fig. 1m), and the expression of cyclin-dependent kinase (CDK) inhibitor p16^INK4a^ (Fig. 1n) and SASP component VCAM1 (Fig. 1o) in aortic ECs. Additionally, DNA damage response, represented by increased γ-H2A.X phosphorylation, emphasized cellular senescence phenotype in ECs from aged aortas (Fig. 1p).

The strong association between age-related PAA elevation, *Clostridium* genus (seen in both aged humans and mice), particularly *Clostridium* sp. ASF356 and vascular dysfunction highlights the key role of gut bacteria in phenylalanine metabolism and its impact on endothelial biology and vascular decline in aging.

### *Clostridium* sp. ASF356 triggers endothelial senescence in vivo

**Fig. 2:**
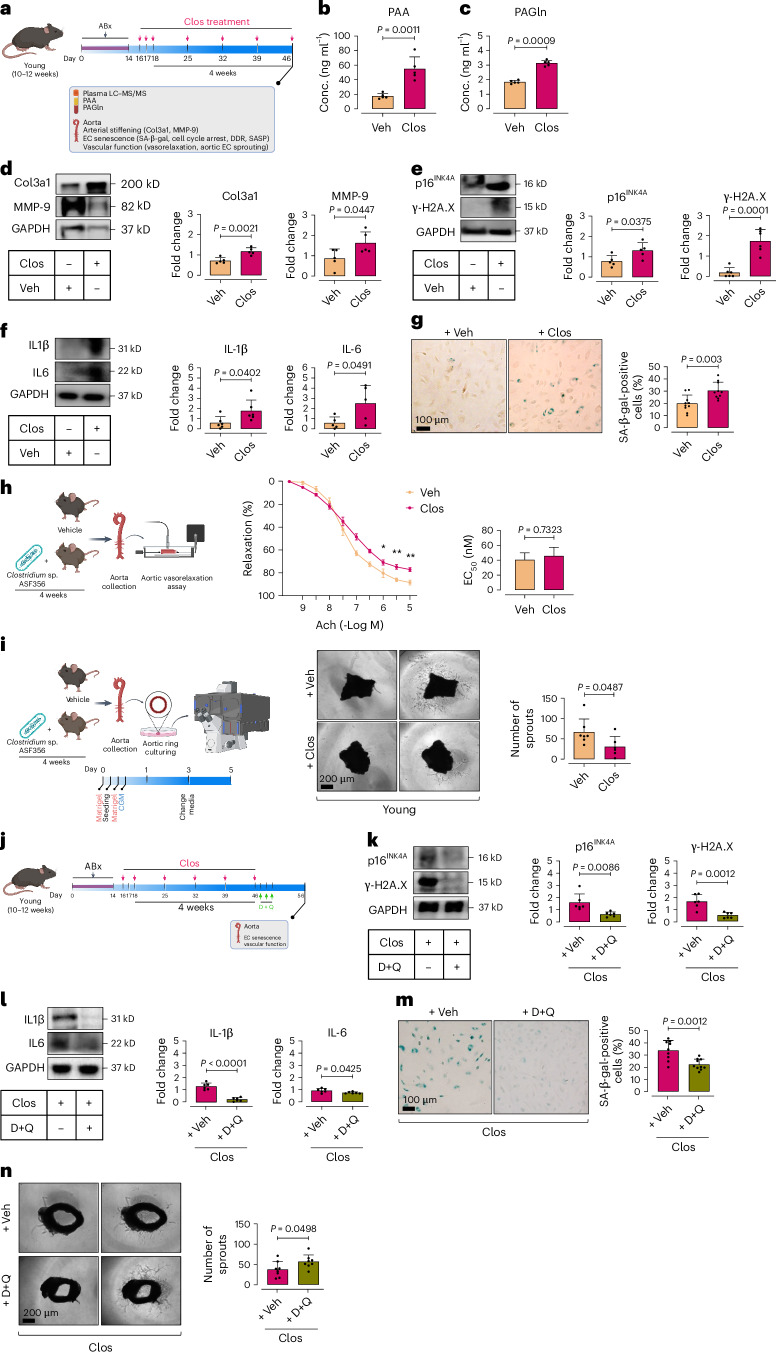
*Clostridium* sp. ASF356 induces EC senescence in aortas of colonized mice. **a**, Schematic of the experimental setting: 10–12-week-old C57BL/6J male mice were pretreated with antibiotics (ABx) for 2 weeks, then colonized with *Clostridium* sp. ASF356 (*Clos*) by oral gavage for 4 weeks. **b**,**c**, LC–MS/MS quantification demonstrates plasma PAA (**b**) and PAGln (**c**) levels in mice (*n* = 5–6). **d**, Immunoblotting represents the expression of arterial stiffening biomarkers collagen 3a1 (Col3a1) and matrix metalloproteinase-9 (MMP-9) in aortas of mice (*n* = 5–6). **e**–**g**, ECs isolated from mouse aortas were subjected to characterization of cellular senescence hallmarks, including proliferative arrest marker p16^INK4A^ and DDR marker γ-H2A.X (**e**), the SASP components IL-1β and IL-6 (**f**) and SA-β-gal activity (**g**) (*n* = 5–6). **h**, Schematic of ex vivo force tension myography. Aortic rings from *Clos*-colonized mice showed impaired vasorelaxation responses (%) to acetylcholine (*n* = 5–6). **i**, Schematic of aortic endothelial sprouting assays. Confocal micrographs of aortic rings illustrate angiogenic capacity of ECs. Quantitative plot for numbers of aortic endothelial sprouts (*n* = 5–6) (right). **j**, Schematic of the experimental setting: 10–12-week-old C57BL/6J male mice underwent ABx pre-treatment (2 weeks) and *Clos* colonization (4 weeks), followed by senolytic dasatinib (D) + quercetin (Q) treatment (D, 5 mg kg^−1;^ Q, 50 mg kg^−1^, daily by oral gavage for 3 consecutive days). **k**–**m**, Senescence hallmarks, including p16^INK4A^ and γ-H2A.X (**k**), IL-1β and IL-6 (**l**) and SA-β-gal activity (**m**), were examined in ECs isolated from aortas of mice (*n* = 6). **n**, Confocal micrographs of aortic rings illustrate the effects of D + Q on aortic endothelial sprouting capacity in *Clos*-colonized mice. Quantitative plot for numbers of endothelial sprouts (*n* = 8) (right). Data were determined in eight micrographs and represent triplicated biologically independent experiments (**i**,**n**). Scale bars, 100 and 200 μm. Error bars represent s.d. (**b**–**g**,**i**,**k**–**n**) or s.e.m. (**h**). *P* values were calculated using a two-tailed unpaired Student’s *t*-test (**b**–**i**,**k**–**n**). Images created with BioRender.com (**a**,**h**–**j**). *P < 0.05, **P < 0.01. Source data

To explore whether *Clostridium* sp. ASF356, by elevating PAA, causally triggers aortic endothelial senescence and dysfunction in young mice, mirroring observations in aged mice, we colonized antibiotics (ABx)-pretreated mice with *Clostridium* sp. ASF356 (*Clos*) via oral gavage for 4 weeks (Fig. 2a). PCR confirmed successful colonization, as evidenced by a significant increase in *GroEL* gene transcription, which is unique and highly specific to the bacterium and encodes the conserved molecular chaperone GroEL (Supplementary Fig. 2a,c). *Clos* colonization led to a ~3.15-fold increase in PAA and ~1.7-fold increase in PAGln in plasma (Fig. 2b,c) compared to vehicle-recipient counterparts, confirming the bacterium’s ability to metabolize phenylalanine to PAA and PAGln in vivo.

Upon tissue collection, we noted diminished aortic elasticity, concomitant with heightened expression of tissue remodeling biomarkers collagen type III (Col3a1) and matrix metalloproteinase-9 (MMP-9) (Fig. 2d). Additionally, we observed increased vascularity of perivascular adipose tissue (PVAT) and visceral adipose tissue wet weight, both linked to poor cardiovascular outcomes and cardiovascular mortality^19^. These observations were corroborated by a senescence-like phenotype in aortic ECs from *Clos*-colonized mice, evidenced by a significant increases in expression of the proliferative arrest marker p16^INK4a^ and DNA damage (γ-H2A.X phosphorylation) (Fig. 2e). The SASP components IL-1β and its downstream effector IL-6 were also upregulated (Fig. 2f), along with increased SA-β-gal positivity (Fig. 2g). These cellular alterations culminated in a marked decline in vasorelaxation responses, demonstrated as significantly decreased maximal relaxation at 10^−5^ M acetylcholine (vehicle, 88.53 ± 5.40; *Clos*, 77.15 ± 6.66; *P* < 0.01), with a nonsignificant rightward shift in EC_50_ (vehicle, 40.57 ± 5.20 nM; *Clos*, 45.77 ± 14.96 nM) (Fig. 2h). Furthermore, angiogenic capacity was reduced, with markedly lower numbers of endothelial sprouts in aortic rings from *Clos*-colonized mice (Fig. 2i).

To confirm whether *Clos* causally promotes EC senescence and dysfunction in vivo, we exploited the potent senolytic D + Q cocktail of 5 mg kg^−1^ dasatinib (D) + 50 mg kg^−1^ quercetin (Q) to mitigate EC senescence in *Clos*-colonized mice (Fig. 2j). Our data show that D + Q effectively reverses major senescence hallmarks, as evidenced by reduced expression of p16^INK4a^ and γ-H2A.X (Fig. 2k), IL-1β and IL-6 (Fig. 2l), and lysosomal SA-β-gal activity (Fig. 2m). This senescence rescue corresponded with restored vascular function, indicated by enhanced endothelial sprouting in aortic rings from treated mice (Fig. 2n). Together, these data provide strong support for a direct contribution of *Clostridium* sp. ASF356 to endothelial senescence in vivo, potentially through enhanced phenylalanine metabolism to PAA.

### PAA causally induces premature endothelial senescence

**Fig. 3:**
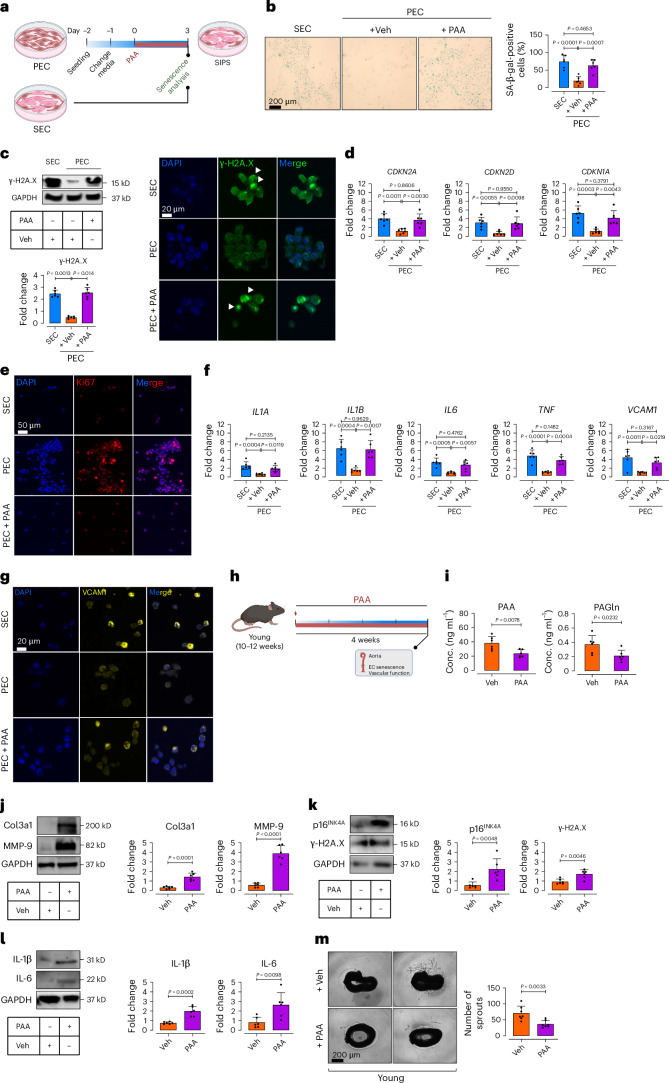
PAA induces endothelial cell senescence. **a**, Schematic diagram of the experimental setting: PECs at p4–5 were treated with PAA (10 μM) for 72 h and then subjected to senescence hallmarks profiling. **b**, SA-β-gal staining shows senescence-associated lysosomal alterations in PECs treated with PAA versus vehicle versus replicative SECs. Quantitative plots are shown for SA-β-gal-positive cells (%) (*n* = 6) (right). **c**, Immunoblots (top) and γ-H2A.X immunostaining (bottom) represent DDR (*n* = 6). **d**, qPCR shows transcriptional changes of CDK inhibitors *CDKN2A*, *CDKN2D* and *CDKN1A* (*n* = 6). **e**, Immunostaining reveals the expression of proliferation marker Ki67 (*n* = 6). **f**, qPCR demonstrates transcriptional alterations of SASP genes *IL1A*, *IL1B*, *IL6*, *TNF* and *VCAM1* (*n* = 6). **g**, VCAM1 immunostaining shows its expression in ECs (*n* = 6). Data from in vitro cellular experiments represent triplicate biologically independent experiments. **h**, Schematic diagram of the experimental setting: 10–12-week-old C57BL/6J male mice were treated with PAA (50 mg kg^−1^) via daily intraperitoneal (i.p.) injections for 4 weeks. Aortas were collected for characterization of EC senescence and angiogenic capacity. **i**, Plasma levels of PAA (left) and PAGln (right) were quantified by LC–MS/MS in mice (*n* = 6). **j**–**l**, Immunoblots represent the expression of tissue remodeling markers Col3a1 and MMP-9 (**j**) and senescence hallmarks including p16^INK4a^ and γ-H2A.X (**k**) and IL-1β and IL-6 (**l**) in mouse aortas (*n* = 6). **m**, Confocal micrographs of aortic rings illustrate angiogenic incompetence in PAA- versus Veh-treated young mice. Quantitative plot represents the number of aortic endothelial sprouts (*n* = 8) (right). Data were determined in eight micrographs and represent triplicated biologically independent experiments. Scale bar, 20, 50 and 200 μm. Error bars represent s.d. (**b**–**d**,**f**,**i**–**m**). *P* values were calculated using one-way ANOVA followed by Tukey’s post hoc test (**b**–**d**,**f**) and a two-tailed unpaired Student’s *t*-test (**j**–**m**). Images created with BioRender.com (**a**,**h**). Source data

To confirm that PAA directly underpins *Clos*-induced endothelial senescence and angiogenic incompetence, we stimulated proliferating human aortic ECs (p.4–5) with exogenous PAA and compared them to replicative senescent ECs (p.15–17) in vitro (Fig. 3a). PAA-treated proliferating ECs (PEC) exhibited the senescence phenotype (including enlarged, flattened and multinucleated appearance of cells) and associated markers, validated as irreversible proliferative arrest (Supplementary Fig. 3) and exacerbated multiple premature senescence-like parameters such as concomitant increases in numbers of SA-β-gal^+^ cells (Fig. 3b) and expression of γ-H2A.X (Fig. 3c) and CDK inhibitors *CDKN2A* (p16^INK4a^), *CDKN2D* (p19^INK4d^) and *CDKN1A* (p21^WAF1/Cip1^) at messenger RNA level (Fig. 3d), comparable to what seen in replicative SECs. Additionally, PAA markedly reduced the levels of Ki67 signals to the extent seen in SECs, confirming cell-cycle arrest in PAA-exposed PECs (Fig. 3e). Our qPCR assays also demonstrated that PAA significantly increases transcriptional levels of the SASP components, including *IL1Α*, *IL1Β*, *IL6*, *TNF* and *VCAM1* (Fig. 3f). The latter was also confirmed by VCAM1 immunostaining (Fig. 3g), mirroring observations in aged aortas (Fig. 1n).

**Extended Data Fig. 2:**
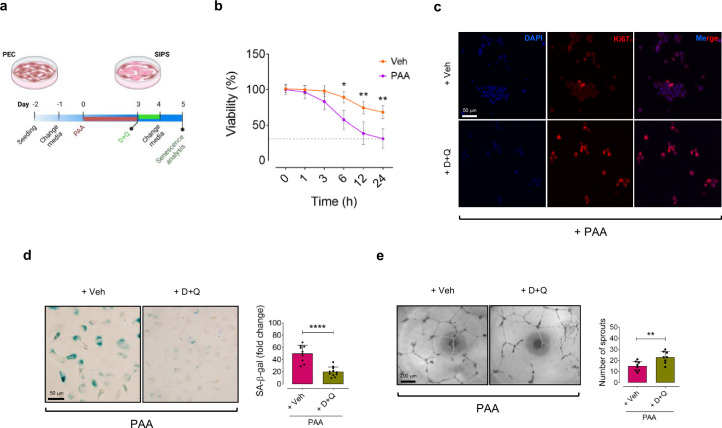
D + Q reduces senescence in PAA-exposed ECs. **a**, Schematic diagram of the experimental setting: PECs were treated with PAA (10 μM, for 72 h) in the presence or absence of Dasatinib+Quercetin (D + Q: 500 nM + 20 μM, for 24 h), followed by assessment of senescence phenotype and tube formation. **b**, Cell counts demonstrate that D + Q significantly reduces the viability percentage (%) of PAA-exposed PECs over Veh-treated PECs. The cells at passages 3 to 5 were characterized as PEC (n = 6). **c,d**, D + Q ameliorates PAA-induced senescence phenotype in HAECs: (**c**) Ki67 immunostaining shows that D + Q markedly increases the number of Ki67^+^ PECs (n = 6); (**d**) Representative images of SA-β-gal staining demonstrates that D + Q significantly decreases the number of SA-β-gal^+^ cells (%) (n = 6). **e**, Confocal images represent the 2D matrigel tube formation of PAA-exposed PECs treated with D + Q or vehicle. Right, quantitative plot shows a significantly higher number of tubes formed by D + Q-treated PECs compared to vehicle counterparts (n = 8). Scale bar, 50 and 200 μm. Experiments were triplicated independently. Statistical analysis was performed with a one-way ANOVA followed by Tukey’s post *hoc* test (**b**) and a two-tailed unpaired Student’s *t*-test (**c, d**). Images created with BioRender.com (**a**). (*P < 0.05, **P < 0.01, ****P < 0.0001). Source data

To further support these findings, we conducted in vitro treatment of PECs with PAA (10 µM, for 72 h), followed by senolytic D + Q (500 nM + 20 μM, for 24 h) (Extended Data Fig. 2a). Our results demonstrate that D + Q selectively reduced viability of senescent ECs (~67.82%), while sparing proliferating cells (~88.55%) (Extended Data Fig. 2b). Consistently, D + Q significantly increased Ki67^+^ cells and reduced numbers of SA-β-gal^+^ cells (~60.12%) (Extended Data Fig. 2c,d). The selective elimination of PAA-induced senescent ECs subsequently improved endothelial tube formation (~1.5-fold), an in vitro measure of angiogenesis (Extended Data Fig. 2e).

As a proof of concept, we investigated senescence-promoting effects of PAA in vivo (Fig. 3h). Our data revealed that a 4-week PAA administration (50 mg kg^−1 ^d^−1^, i.p.) led to a significant rise in circulating PAA and PAGln levels (Fig. 3i), accompanied by upregulated stiffening markers Col3a1 and MMP-9 (Fig. 3j) in aortas of young mice, mirroring findings in *Clos*-colonized mice (Fig. 2d). Notably, cellular senescence markers were upregulated in aortic cells, evidenced by significant increases in of p16^INK4a^ and γ-H2A.X (Fig. 3k), alongside IL-1β and IL-6 (Fig. 3l) in aortic ECs. The senescent phenotype was associated with impaired angiogenic capacity compared to vehicle-recipient controls (Fig. 3m). Collectively, these findings support a causal role for PAA in driving EC senescence, akin to aging-related endothelial decline.

### PAA induces mitochondrial dysfunction via excess H_2_O_2_

**Fig. 4:**
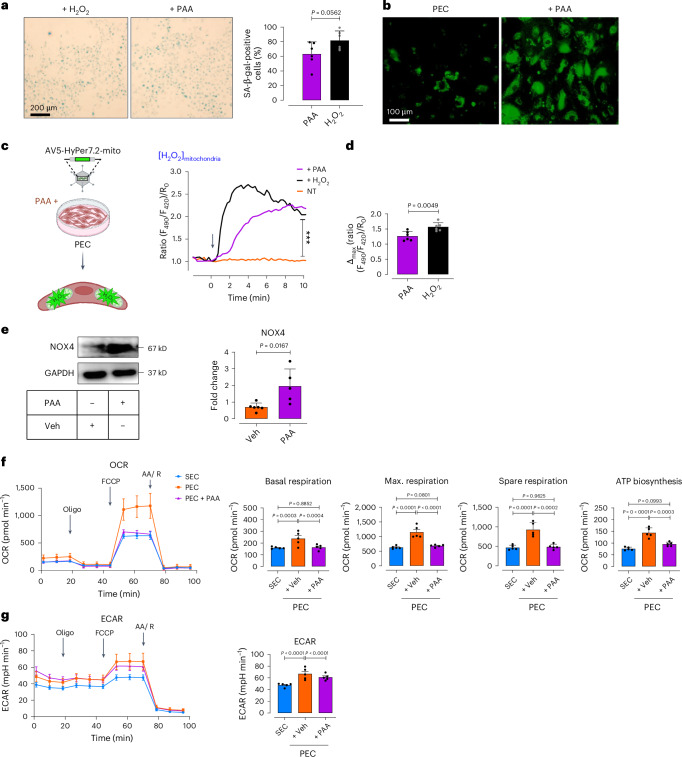
PAA regulates premature EC senescence by H_2_O_2_-mediated mitochondrial impairment. **a**, SA-β-gal staining shows senescence-associated lysosomal alterations in Veh-treated PECs compared to PECs incubated with exogenous H_2_O_2_ (50 μM). Quantitative plots for SA-β-gal-positive cells (%) are shown (*n* = 6). **b**, CellRox Green staining reveals the generation of intracellular ROS, including H_2_O_2_, (as green fluorescence) in the presence or absence of PAA. **c**, Schematic diagram of the experimental setting: PECs were transduced with adenovirus 5 (AV5)-HyPer7.2 targeted to the cell mitochondria for further ratiometric fluorescence imaging to detect mitochondrial H_2_O_2_ generation in the presence of PAA. Right, the curves demonstrate significantly higher H_2_O_2_ responses in the mitochondria of HyPer7.2- transfected PECs following treatment with either PAA (*n* = 25) or exogenous H_2_O_2_ (*n* = 27) compared to vehicle (*n* = 24). **d**, Bar chart shows maximal mitochondrial H_2_O_2_ responses to PAA or exogenous H_2_O_2_ (*n* = 6). **e**, Immunoblot analysis demonstrates the expression of NOX4 in PECs (*n* = 5–6). **f**, Mitochondrial respiratory rates in PECs treated with either PAA or vehicle and in replicative SECs were measured using Seahorse flux analyzer by Cell Mito Stress kit (*n* = 5). Bar charts reveal oxygen consumption rate (OCR) represented by maximal, basal, and spare reserve, and ATP biosynthesis in PAA-exposed PECs compared to PECs incubated with vehicle. **g**, Glycolysis was measured by ECARs in vehicle- or PAA-treated PECs and replicative SECs under basal conditions (*n* = 5). Bar chart depicts ECAR in Veh-treated PECs compared to PAA-exposed PECs or replicative SECs (right). Data from in vitro cellular experiments represent triplicate biologically independent experiments. Scale bars, 100 and 200 μm. Error bars represent s.d. (**a**,**d**–**g**) or s.e.m. (**c**,**f**,**g**). *P* values were calculated using a two-tailed unpaired Student’s *t*-test (**a**,**e**), two-tailed Mann–Whitney *U*-test (**d**) and one-way ANOVA followed by Tukey’s post hoc test (**c**,**f**,**g**). Images created with BioRender.com (**c**). ***P < 0.001. Source data

To better understand the mechanism underpinning PAA-induced EC senescence, we observed that PAA triggers premature EC senescence to the magnitude seen in response to exogenous H_2_O_2_ (Fig. 4a). Since excessive ROS, particularly H_2_O_2_, are well characterized to trigger EC senescence^6^, we tested whether PAA increases ROS generation. CellRox Green fluorescence imaging confirmed markedly higher global ROS generation in PAA-exposed PEC (Fig. 4b). Using the ultrasensitive H_2_O_2_ biosensor, HyPer7.2, we detected significant elevation of mitochondrial H_2_O_2_ in presence of PAA (Fig. 4c). As shown in Fig. 4d, maximal HyPer7.2 ratio in PAA-treated PECs was comparable to that seen with exogenous H_2_O_2_ (50 μM). To identify the source of excess mitochondrial H_2_O_2_, we found that PAA-treated PECs exhibit increased NADPH oxidase 4 (NOX4) expression (Fig. 4e). Meanwhile, glutathione peroxidase (*Gpx1*) transcripts were markedly reduced upon exposure to PAA (‘stress condition’), counteracting antioxidant defense (Supplementary Fig. 4).

Altered metabolism is a key driver of endothelial senescence^20^, so we tested mitochondrial function, impairment of which aggravates oxidative stress. Our Seahorse mitochondrial stress assay displayed ~twofold reduction in basal and maximal respiration rates in PAA-exposed PECs to the magnitude seen in SECs (Fig. 4f). Moreover, spare respiratory capacity, with FCCP-stimulated oxidative phosphorylation (OXPHOS) uncoupling, along with ATP biosynthesis was impaired in response to PAA (Fig. 4f). In agreement with reduced mitochondrial respiration, PAA markedly reduced basal extracellular acidification rate (ECAR), indicating glycolytic decline (Fig. 4g), a major energy supplier in ECs^21^.

### PAA triggers endothelial senescence by epigenetic alterations

Senescence involves extensive epigenetic reprogramming, including histone modifications that alter gene expression patterns^4,22^. Histone deacetylase 4 (HDAC4) is an epigenetic regulator of cellular senescence^23^, modulated by post-translational modification partly regulated by NOX4-derived H_2_O_2_ in ECs^24^. Accordingly, we tested whether PAA, which earlier was shown to induce mitochondrial H_2_O_2_ generation (Fig. 4b–d), impacts HDAC4 and epigenetic responses, rendering EC senescence.

**Fig. 5:**
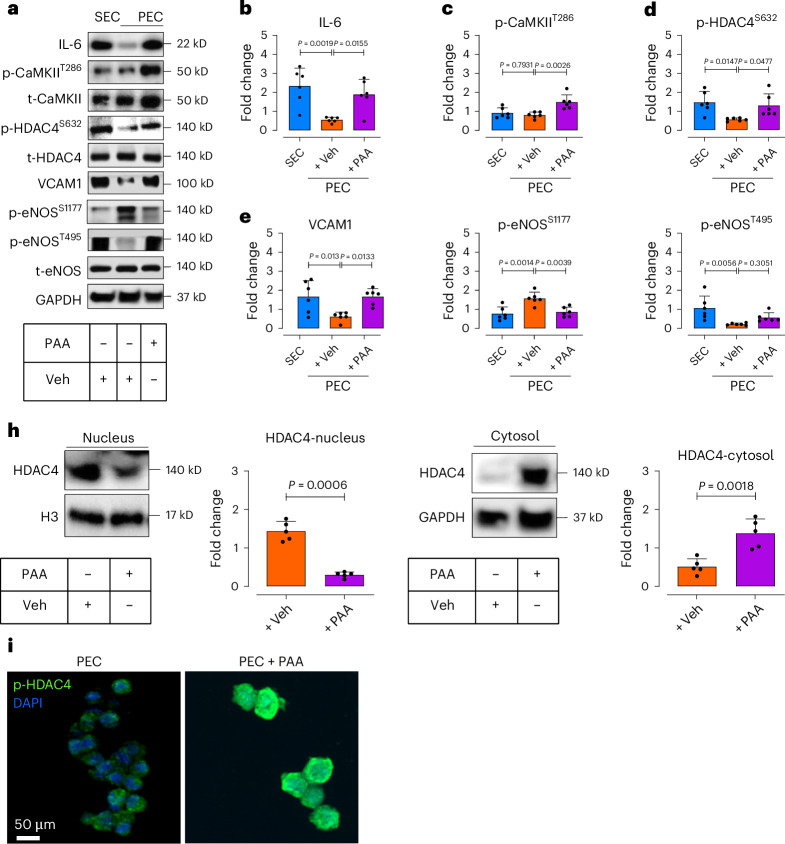
PAA induces EC senescence through the SASP-epigenetic regulation. **a**–**g**, Immunoblotting represents the expression of IL-6 (**b**), CaMKII-mediated phosphorylation of HDAC4 at Ser632 (**c**,**d**), VCAM1 expression (**e**), post-transcriptional modifications of eNOS at Ser1177 and Thr495 (**f**,**g**) in response to PAA in PECs (*n* = 6). **h**, Immunoblots and quantitative plots reveal translocation of HDAC4, represented as decreased expression in the nucleus (left) and increased expression in the cytosol (right) in PECs in response to PAA (*n* = 5). **i**, p-HDAC4 immunostaining illustrates HDAC4 phosphorylation, facilitating its translocation toward the cell cytosol in PECs exposed to PAA (*n* = 6). Scale bar, 50 μm. Data from in vitro cellular experiments represent triplicate biologically independent experiments. Error bars represent s.d. (**b**–**h**). *P* values were calculated using one-way ANOVA followed by Tukey’s post hoc test (**b**–**g**) and a two-tailed unpaired Student’s *t*-test (**h**). Source data

**Extended Data Fig. 3:**
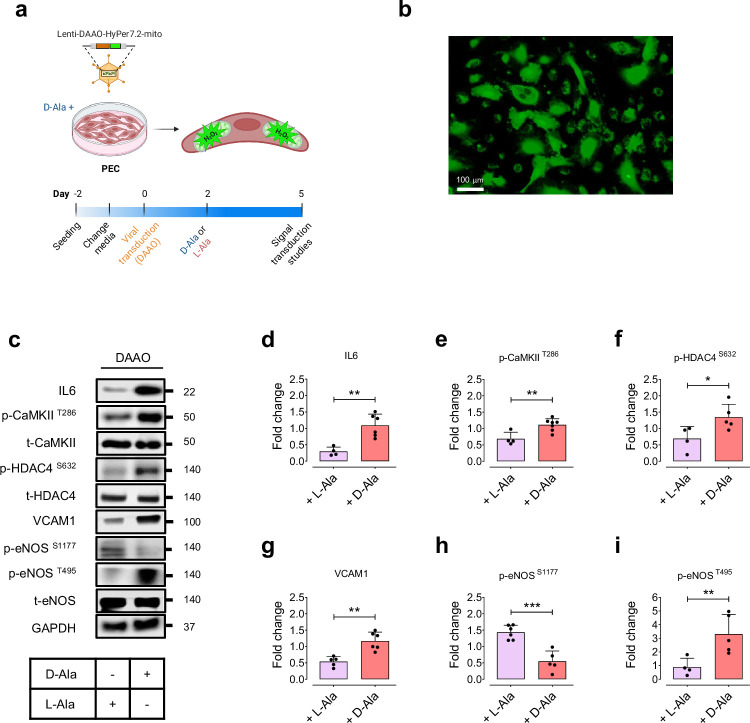
Chemogenetic mitochondrial H_2_O_2_ regulates endothelial NO signaling through IL-6-HDAC4 pathway. **a**, Schematic diagram of the experimental setting: PECs were transduced with lentiviral vectors encoding D-amino acid oxidase (DAAO)-HyPer7.2 targeted to the cell mitochondria for generation of mitochondrial H_2_O_2_ in the presence of D-alanine (10 mM) for 72 h. **b**, Representative widefield ratiometric HyPer7.2 images of PECs transduced with Lenti-DAAO-HyPer7.2-mito constructs. **c-i**, Immunoblots (**c**) and quantitative plots show that chemogenetic mitochondrial H_2_O_2_, produced by DAAO in response to D-alanine but not L-alanine, orchestrates IL-6-mediated CaMKII-HDAC4 phosphorylation and the subsequent VCAM1-regulated eNOS phosphorylation at Ser1177 and Thr495 in PECs (n = 4–6). Scale bar, 100 μm. Error bars represent SD (**d–i**). Data represent triplicated biologically independent experiments. *P* values were calculated using a two-tailed unpaired Student’s *t*-test (**d**–**i**). Images created with BioRender.com (**a**). (*P < 0.05, **P < 0.01, ***P < 0.001). Source data

**Extended Data Fig. 4:**
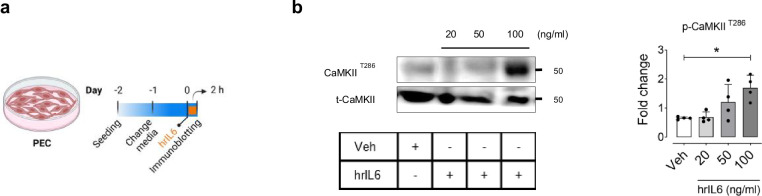
hrIL6 regulates CaMKII post-translation modification in ECs. **a**, Schematic diagram of the experimental setting: PECs were treated with the human recombinant IL-6 (hrIL6) at specific concentrations (0, 20, 50, 100 ng/ml) for 2 h. **b**, Immunoblots (Left) and quantitative plots (Right) demonstrate that hrIL6 dose-dependently increases CaMKII phosphorylation at Thr286 in PECs (n = 6). Error bars represent SD (**b**). Experiments were triplicated independently. *P* values were calculated using one-way ANOVA followed by Tukey’s post *hoc* test (**b**). Images created with BioRender.com (**a**). (***P < 0.001). Source data are provided as a Source Data file. Source data

**Extended Data Fig. 5:**
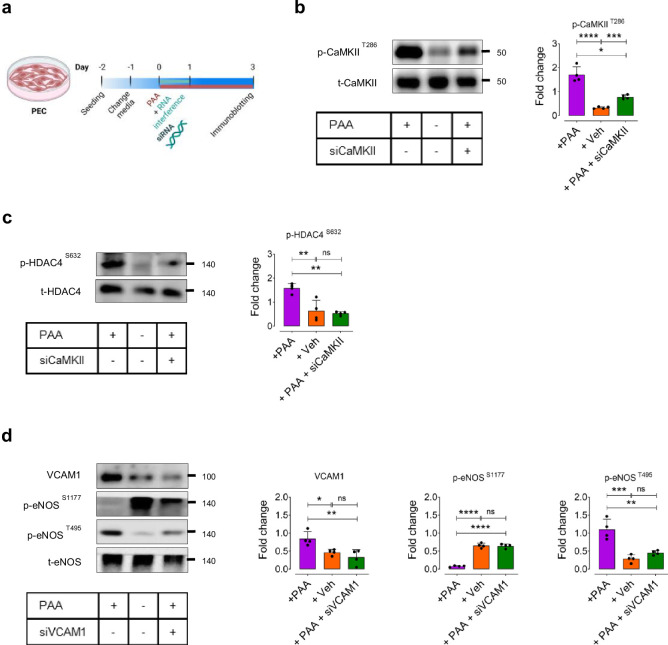
CaMKII-mediated SASP pathway underlies PAA-induced EC dysfunction. **a**, Schematic diagram of the experimental setting: PECs were transfected with siCaMKII or siVCAM1 (for 24 h) in the presence or absence of PAA (10 μM, for 72 h). **b, c**, Immunoblots (left) and quantitative plots (right) characterizing the effects of PAA on the PAA-induced post-translational modifications of CaMKII (**b**) and its downstream epigenetic regulator HDAC4 (**c**) using CaMKII knockdown approach in PECs (n = 4). **d**, Analysis of VCAM1 expression and eNOS phosphorylation at Ser1177 and Thr495 in siVCAM1-transfected PECs in the presence or absence of PAA (n = 4). Error bars represent SD (**b–d**). Data represent triplicated biologically independent experiments. *P* values were calculated using one-way ANOVA followed by Tukey’s post *hoc* test (**b–d**). Images created with BioRender.com (**a**). (*P < 0.05, **P < 0.01, ***P < 0.001, ****P < 0.0001, ns, not significant). Source data are provided as a Source Data file. Source data

**Extended Data Fig. 6:**
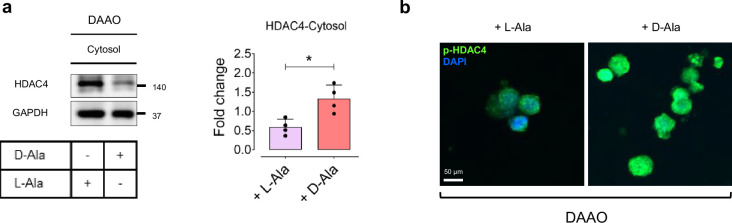
H_2_O_2_ orchestrates HDAC4 phosphorylation and its nuclear export. **a**, Immunoblots (Left) and quantitative plot (Right) reveal HDAC4 unclear export, represented as an increased expression in the cytosol in response to intracellular H_2_O_2_ generated by DAAO in the presence of D-alanine (10 mM, for 72 h) in PECs (n = 4). **b**, p-HDAC4 immunostaining shows that intracellular H_2_O_2_ markedly increases HDAC4 phosphorylation that facilitates its translocation towards the cell cytosol in PECs (n = 6). Scale bar, 50 μm. Error bars represent SD (**a**). Data represent triplicated biologically independent experiments. *P* values were calculated using a two-tailed unpaired Student’s *t*-test (**a**). (*P < 0.05). Source data are provided as a Source Data file. Source data

In line with Fig. 3f, PAA significantly increases IL-6 expression at a protein level in PECs, comparable to those in SECs (Fig. 5a,b). As shown in Extended Data Fig. 3a–f, chemogenetic H_2_O_2_ (by d-amino acid oxidase (DAAO) in presence of d-alanine^25^) upregulates IL-6, confirming that PAA-mediated SASP is H_2_O_2_-dependent. As CaMKII is a both H_2_O_2_- and IL-6-responsive protein kinase that regulates HDAC4 phosphorylation^24^, we assessed how PAA regulates the CaMKII–HDAC4 pathway. Our findings revealed that PAA significantly increases phosphorylation of CaMKII at Thr286 and its downstream HDAC4 at Ser632 in PECs (Fig. 5a,c,d). As a proof of concept, treatment of PECs with human recombinant IL-6 (hrIL-6) replicated these effects, confirming PAA’s role in IL-6-CaMKII activation (Extended Data Fig. 4a,b). Using siRNA against CaMKII, we observed a marked reduction in HDAC4^S632^ phosphorylation in PAA-exposed PECs (Extended Data Fig. 5a–c). Increased HDAC4 phosphorylation facilitates its nuclear export toward the cytosol, evidenced by lower nuclear HDAC expression rather than the cytosolic form (Fig. 5h). Our immunostaining confirms that PAA elicits cytosolic localization of HDAC4 (Fig. 5i), as similarly seen in response to intracellular H_2_O_2_ generated by DAAO (Extended Data Fig. 6a,b).

**Extended Data Fig. 7:**
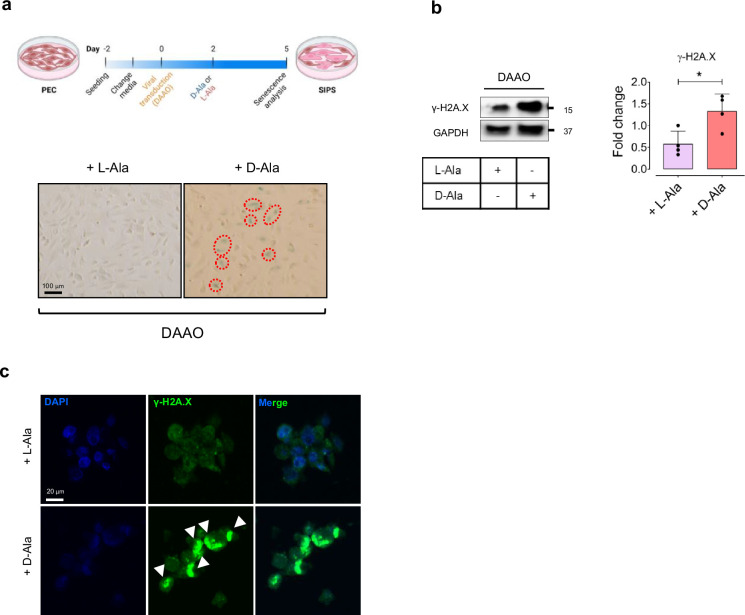
Chemogenetic H_2_O_2_ promotes EC senescence. **a**, Schematic diagram of the experimental setting: PECs were transduced with lentiviral vectors encoding D-amino acid oxidase (DAAO) targeted to the cell mitochondria for generation of mitochondrial H_2_O_2_ in the presence D-alanine (10 mM) for 72 h. Representative images of SA-β-gal staining demonstrates that DAAO-mediated mitochondrial H_2_O_2_ induced cellular senescence in PECs, as shown by increased SA-β-gal-positive cells (%) (n = 6). **b,c**, Immunoblots (**b**) and γ-H2A.X immunostaining images (**c**) reveal a marked increase in DDR following mitochondrial H_2_O_2_ production in DAAO-transduced PECs incubated with D-alanine (n = 4–6). Scale bar, 20 and 100 μm. Red circles indicate SA-β-gal-positive cells. Error bars represent SD (**b**). Data represent triplicated biologically independent experiments. *P* values were calculated using a two-tailed unpaired Student’s *t*-test (**b**). Images created with BioRender.com (**a**). (*P < 0.05). Source data are provided as a Source Data file. Source data

One major downstream of HDAC4 contributing to endothelial dysfunction is the SASP component VCAM1 (ref. ^26^). Our findings demonstrated that PAA triggers VCAM1 expression at both mRNA (Fig. 3f) and protein (Fig. 5a,e) levels in PECs. Furthermore, PAA-exposed PECs exhibited reduced eNOS^Ser1177^ phosphorylation (Fig. 5a,f), whereas phosphorylation at Thr495 was robustly increased (Fig. 5a,g). To confirm VCAM1’s role in regulating eNOS signaling, siVCAM1 transfection of PAA-treated PECs showed that VCAM1 silencing significantly restores eNOS activation (phosphorylation at Ser1177) (Extended Data Fig. 5d). These confirm causal contribution of PAA to endothelial dysfunction through VCAM1 modulation. To test deeply whether H_2_O_2_ itself underpins PAA-mediated VCAM1-eNOS regulation, we exploited our chemogenetic DAAO model to induce mitochondrial H_2_O_2_ in PECs. DAAO-generated H_2_O_2_ in cell mitochondria led to increased VCAM1 expression and suppression of the nitric oxide (NO) signaling axis by eNOS^Ser1177^ dephosphorylation and increased phosphorylation of eNOS^Thr495^ (Extended Data Fig. 3g–i). Similar to outcomes of PAA, chemogenetic H_2_O_2_ also increased numbers of SA-β-gal^+^ cells (Extended Data Fig. 7a) and γ-H2A.X phosphorylation (Extended Data Fig. 7b,c), confirming H_2_O_2_ as a driver of PAA-induced endothelial senescence. Our signal transduction studies conclude that PAA causally induces mitochondrial H₂O₂ overproduction, triggering IL-6-mediated HDAC4 cytosolic translocation, which upregulates VCAM1. PAA subsequently disrupts eNOS signaling, leading to vascular dysfunction (Supplementary Fig. 5).

### PAA impairs angiogenic capacity of endothelial cells

**Fig. 6:**
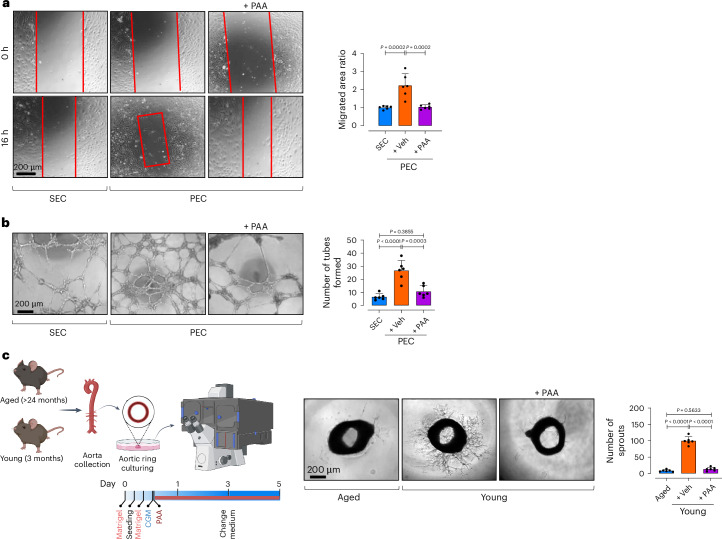
PAA reduces EC proliferation and angiogenesis. **a**, Confocal micrographs depict cell migration, represented as areas of uncovered surface, in PECs treated with PAA or vehicle and in replicative SECs. Bar chart represents the ratio of cell migrated area (*n* = 6) (right). **b**, Confocal images represent two-dimensional Matrigel tube formation of ECs. Quantitative plot shows numbers of tubes formed by ECs (*n* = 6) (right). **c**, Confocal micrographs of aortic rings from aged and young mice represent angiogenic capacity in response to PAA or vehicle. Quantitative plot is shown for numbers of aortic endothelial sprouts (*n* = 6) (right). Error bars represent s.d. (**a**–**c**). *P* values were calculated using one-way ANOVA followed by Tukey’s post hoc test (**a**–**c**). Image created with BioRender.com (**c**). Source data

Because endothelial senescence is linked to impaired angiogenesis, we reasoned that PAA confers critical effects on endothelial angiogenic capacity. Our results show markedly lower migration rates (~twofold) in PAA-treated PECs at 16 h post-scratch (Fig. 6a). Tube formation assay also demonstrated a ~fourfold reduction in numbers of tubes formed following PAA treatment, indicating compromised angiogenic capacity (Fig. 6b). In agreement with these data, aortic rings from young mice, incubated with PAA, exhibited markedly attenuated endothelial sprouting, mirroring aged aortas (Fig. 6c). Sprouted aortic rings were also immunostained for endothelial-specific adhesion molecules VE-cadherin (VEC) and CD31. Imaging data confirm a significant reduction in total volume of sprouted VEC^+^, CD31^+^ ECs in response to PAA (Supplementary Fig. 6).

These findings highlight PAA as a key driver of endothelial angiogenic incompetence, mediated through diverse senescence-promoting mechanisms, including SASP, epigenetic modifications and energy imbalance (Supplementary Fig. 7).

### Acetate rescues PAA-induced endothelial senescence as a senomorphic

**Fig. 7:**
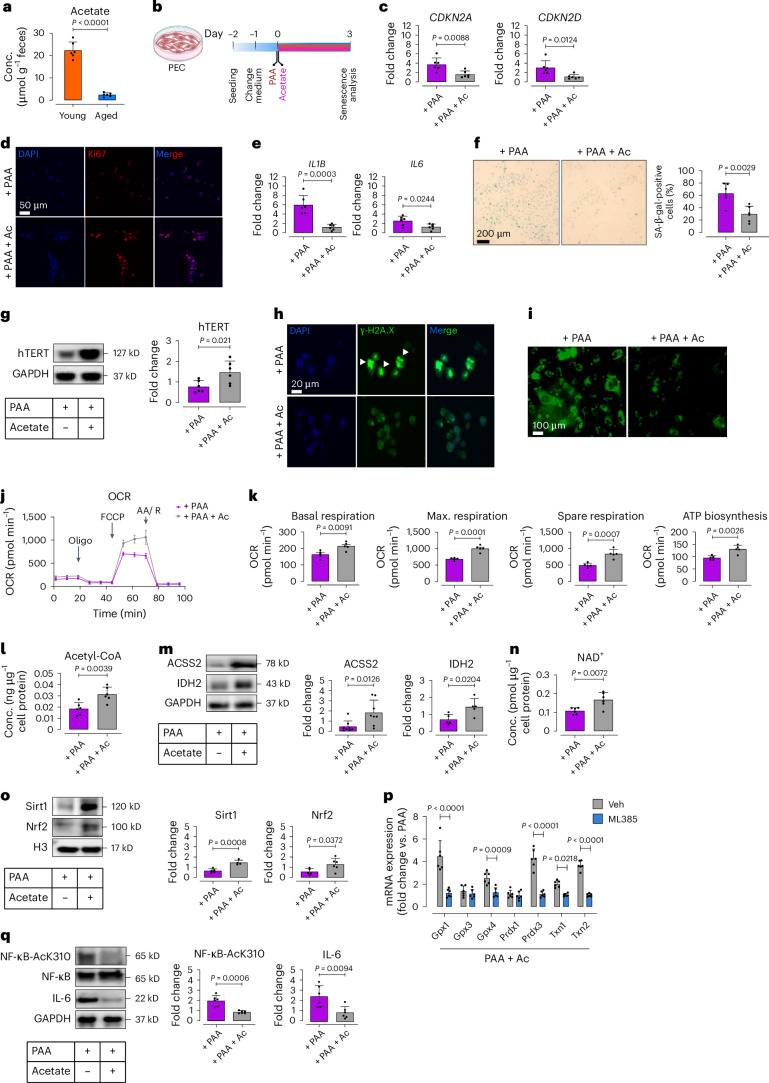
Acetate rescues PAA-induced EC senescence through senomorphic effects. **a**, Bar charts represent fecal acetate levels in aged mice compared to young mice, as quantified by HPLC-RI (*n* = 6). **b**, Schematic of experimental setting. PECs were co-treated with PAA (10 μM) and sodium acetate (3 μM) for 72 h, followed by senescence analysis. **c**, qPCR analyses reveal transcriptional changes of CDK inhibitors *CDKN2A* and *CDKN2D* (*n* = 6). **d**, Ki67 immunostaining represents its expression (*n* = 6). **e**, Bar charts represent transcriptional changes of the SASP components *IL1Β* and *IL6*, as assessed by qPCR (*n* = 6). **f**, Representative images of SA-β-gal staining (left) and bar chart (right) demonstrate senescence-associated lysosomal alterations (*n* = 6). **g**, Immunoblots reveal the expression of hTERT, indicating telomere length restoration (*n* = 6). **h**, Representative immunostaining images show γ-H2A.X foci, as a DDR marker (*n* = 6). **i**, CellRox Green staining demonstrates intracellular ROS generation (*n* = 6). **j**,**k**, Seahorse bioenergetic analysis reveals mitochondrial OCR in PAA- versus acetate + PAA-treated PECs. Bar charts show basal and maximal OCR, spare reserve, and ATP production (*n* = 5). **l**, Fluorometric assay reveals cellular acetyl-coA concentrations (*n* = 6). **m**, Immunoblots represent the expression of acetyl-coA synthetase 2 (ACSS2) and isocitrate dehydrogenase 2 (IDH2) (*n* = 5–8). **n**, Bar charts demonstrate NAD^+^ levels, as quantified by colorimetric assay (*n* = 6). **o**, Immunoblots represent the expression of NAD^+^-dependent Sirt1 and the redox regulator Nrf2 (*n* = 5). **p**, qPCR analysis reveals transcription of the Nrf2-regulated antioxidant enzymes glutathione peroxidase 1 and 4 (*Gpx1* and *Gpx4*), peroxiredoxin 3 (*Prdx3*), and thioredoxin 1 and 2 (*Txn1* and *Txn2*) in PAA + sodium acetate-exposed PECs in the presence or absence of the pharmacological Nrf2 inhibitor, ML385 (5 μM for 24 h) (*n* = 6). **q**, Immunoblots demonstrate the acetylation level of the heterodimeric subunit of NF-κB at Lys310, along with the expression of its downstream inflammatory cytokine IL-6 (*n* = 6). Scale bars, 20, 50 and 100 μm. Error bars represent s.d. (**a**,**c**,**e**–**g**,**k**–**q**) or s.e.m. (**j**,**k**). Data represent triplicate biologically independent experiments. *P* values were calculated using a two-tailed unpaired Student’s *t*-test (**a**,**c**,**e**–**g**,**k**–**o**,**q**) and one-way ANOVA followed by Tukey’s post hoc test (**p**). Image created with BioRender.com (**b**). Source data

Aging disrupts gut microbiota balance toward detrimental metabolites and reduction in health-promoting SCFAs^15,27^ such as acetate, which may counteract vascular senescence. Corroborating our previous findings^15^, fecal acetate levels were markedly lower in aged mice compared to young counterparts (Fig. 7a). To test acetate’s anti-senescence potential, we treated PAA-exposed PECs with sodium acetate (3 μM, for 72 h) (Fig. 7b). As anticipated, acetate rescued proliferation, evidenced by significant reduction in *CDKN2A* and *CDKN2D* transcripts (Fig. 7c) and increase in Ki67 signals (Fig. 7d). Additionally, sodium acetate suppressed SASP components *IL1Β* and *IL6* (Fig. 7e), triggered by PAA. Beyond its senomorphic effects, sodium acetate mitigated other senescence hallmarks, shown as reduced SA-β-gal^+^ cells (Fig. 7f), and reversed senescence-associated morphological transformations (Supplementary Fig. 3b). Sodium acetate further counteracted PAA-induced telomere shortening by upregulating hTERT (telomerase reverse transcriptase) expression (Fig. 7g), while reducing DNA damage by γ-H2A.X phosphorylation (Fig. 7h).

To investigate the potential effects of acetate on intracellular ROS sources driving senescence in PAA-exposed ECs, we also examined whether acetate could attenuate mitochondrial ROS production, and through this, restore mitochondrial function. These findings hold notable therapeutic relevance for acetate to mitigate oxidative stress-induced EC dysfunction. Sodium acetate prevented mitochondrial oxidative stress, evidenced by lower numbers of CellRox^+^ cells in presence of PAA (Fig. 7i). Consistently, sodium acetate improved mitochondrial dysfunction, shown as ~30–40% increase in basal and maximal respiration rates and spare respiratory capacity (Fig. 7j,k). Moreover, ATP was biosynthesized at a significantly higher level in PAA-exposed PECs when supplemented with sodium acetate (Fig. 7k). Notably, acetate serves as a crucial precursor for acetyl-CoA, sustaining the TCA cycle and maintaining redox homeostasis^17^. Disruption of this balance by excessive ROS can deplete NAD^+^ levels, and compromise vasculoprotective metabolites^28^. Acetate effectively alleviates mitochondrial oxidative stress (Fig. 7i) and restores energy homeostasis (Fig. 7j,k), accompanied by increased conversion of acetate into acetyl-CoA (Fig. 7l) through activation of acetyl-CoA synthetase (ACSS2; Fig. 7m). Mitochondrial enzymes, including isocitrate dehydrogenase 2 (IDH2), are also upregulated (Fig. 7m) as part of mitochondrial adaptation. While IDH2 contributes to NAD^+^ consumption to generate NADH, acetate-driven enhancement of oxidative phosphorylation (Fig. 7j,k), creates a feedback loop where NADH is rapidly oxidized to regenerate NAD^+^ (Fig. 7n and Supplementary Fig. 8). As a proof of concept, pharmacological inhibition of mitochondrial complex I with rotenone reduced acetate-driven NAD^+^ elevation, confirming its dependence on electron transport chain (ETC) activity (Supplementary Fig. 9a,b). The resulting NAD^+^ boost could activate Sirt1, a key NAD^+^-dependent deacetylase, which plays a central role in mitigating oxidative damage and SASP suppression^28^.

**Extended Data Fig. 8:**
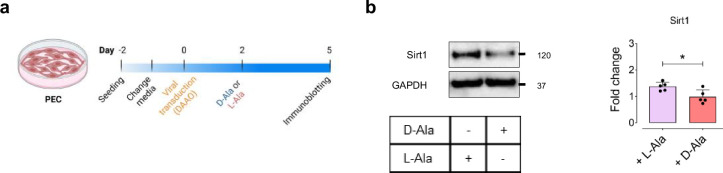
Mitochondrial H_2_O_2_ suppresses Sirt1 in ECs. **a**, Schematic diagram of the experimental setting: PECs were transduced with lentiviral vector encoding DAAO-mito, followed by treatment with D-alanine (10 mM) for 72 h for generation of H_2_O_2_ in cell mitochondria. **b**, Immunoblots (Left) and quantitative plots (Right) demonstrate that mitochondrial H_2_O_2_ significantly reduces Sirt1 expression in PECs (n = 5–6). Error bars represent SD (**b**). Data represent triplicated biologically independent experiments. *P* values were calculated using a two-tailed unpaired Student’s *t*-test (**b**). Images created with BioRender.com (**a**). (*P < 0.05). Source data are provided as a Source Data file. Source data

**Extended Data Fig. 9:**
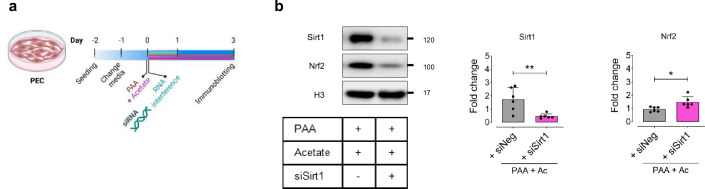
Acetate regulates Nrf2 by Sirt1 modulation in ECs. **a**, Schematic diagram of the experimental setting: PECs were transfected with siNeg or siSirt1 (for 24 h) in the presence of PAA plus sodium acetate (10 μM + 3 μM, for 72 h). **b**, Immunoblots (Left) and quantitative plots (Right) show that silencing of Sirt1 (by siSirt1) significantly reduces the expression of Sirt1 and Nrf2 (deacetylated form) in the nuclei of PECs treated with PAA plus sodium acetate compared to those transfected with siNeg (n = 6). Error bars represent SD (**b**). Data represent triplicated biologically independent experiments. *P* values were calculated using a two-tailed unpaired Student’s *t*-test (**b**). Images created with BioRender.com (**a**). (*P < 0.05, **P < 0.01). Source data are provided as a Source Data file. Source data

**Extended Data Fig. 10:**
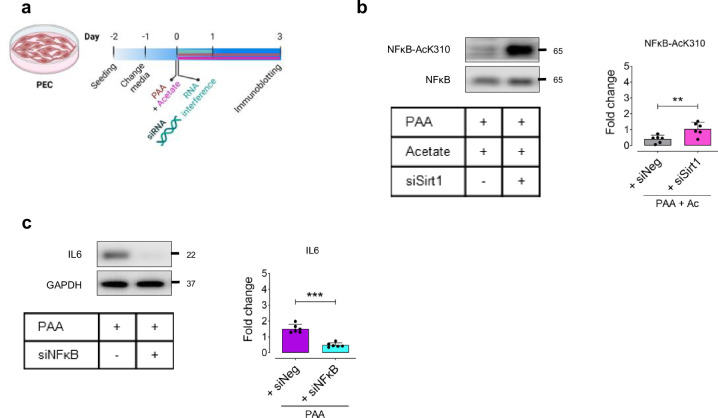
Acetate-mediated SASP downregulation is dependent on Sirt1 in ECs. **a**, Schematic diagram of the experimental setting: PECs were transfected with siNeg, siSirt1, or siNFκB (for 24 h) in the presence of PAA plus sodium acetate (10 μM + 3 μM, for 72 h) or PAA alone (10 μM, for 72 h). **b**, Immunoblots (top) and quantitative plots (bottom) demonstrate that Sirt1 knockdown markedly increases NFκB acetylation at Lys310 in PECs incubated with PAA plus sodium acetate compared to siNeg-transfected counterparts (n = 6). **c**, Immunoblots show that the SASP component IL-6 is downregulated in siNFκB-transfected PECs in the presence of PAA (n = 6). Error bars represent SD (**b,c**). Data represent triplicated biologically independent experiments. *P* values were calculated using a two-tailed unpaired Student’s *t*-test (**b,c**). Images created with BioRender.com (**a**). (**P < 0.01, ***P < 0.001). Source data are provided as a Source Data file. Source data

Our mechanistic studies revealed that sodium acetate both mitigates oxidative stress and dampens SASP induced by PAA. Sodium acetate exerts antioxidant effects through Sirt1-mediated stabilization of nuclear factor erythroid 2-related factor 2 (Nrf2), a master regulator of an array of antioxidant enzymes^6^. PAA alone suppressed Sirt1 and its downstream Nrf2 (Extended Data Fig. 8a,b), likely through mitochondrial H_2_O_2_-induced oxidative stress, while acetate restored nuclear Nrf2 expression in these cells (Fig. 7o). Our loss-of-function studies highlighted that acetate’s role in Nrf2 translocation is Sirt1-dependent, as siSirt1-transfected PAA-PECs showed reduced nuclear Nrf2 (deacetylated form) expression (Extended Data Fig. 9a,b). Acetate-mediated nuclear Nrf2 retention secures its binding to antioxidant responsive elements (AREs), upregulating mitochondrial ROS-neutralizing enzymes *Gpx1*, *Gpx4*, *Prdx3*, *Txn1* and *Txn2*, while pharmacological Nrf2 inhibitor ML385 (5 μM for 24 h) markedly attenuated acetate’s effects in PECs (Fig. 7p). Beyond redox regulation, sodium acetate modulated SASP responses by upregulating Sirt1-mediated epigenetic responses, which suppressed NF-κB signaling. Sodium acetate specifically facilitated Sirt1 recruitment, leading to reduced NF-κB RelA/p65 acetylation at Lys310, thereby repressing target gene encoding the SASP component IL-6 in PAA-exposed PECs (Fig. 7q). Sirt1 silencing reversed this effect, confirming its dominant role in NF-κB deacetylation (Extended Data Fig. 10a,b). As a proof of concept, RelA/p65 knockdown suppressed IL-6 expression, validating NF-κB’s role in SASP regulation (Extended Data Fig. 10c).

Together, these findings identify sodium acetate as a potent senomorphic agent that mitigates PAA-induced endothelial dysfunction through a mechanism distinct from PAA’s mechanism in HDAC4-regulated SASP activation. Sodium acetate exerts its effects on redox homeostasis and SASP by restoring NAD^+^-Sirt1 homeostasis, enhancing Nrf2-driven antioxidant defense and suppressing NF-κB-mediated SASP in the presence of PAA (Supplementary Fig. 10).

### Acetate reverses PAA-induced angiogenic incompetence

**Fig. 8:**
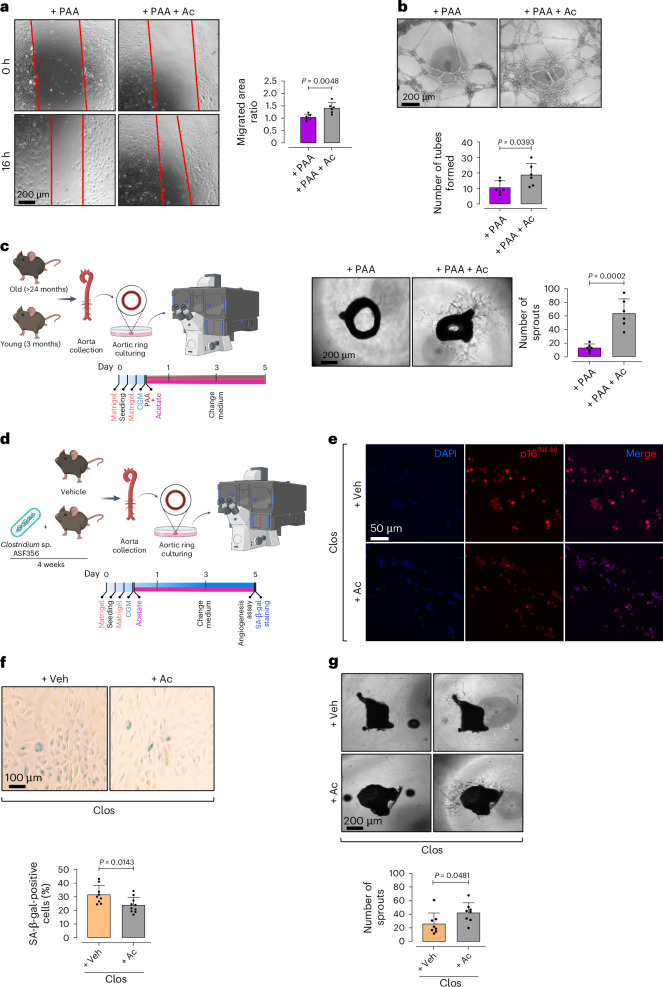
Acetate rescues PAA-induced angiogenic incompetence. **a**–**c**, Confocal micrographs depict the effects of sodium acetate on endothelial angiogenic capacity, represented by cell migration (**a**), tube formation (**b**) and aortic endothelial sprouting (**c**). Quantitative plots represent migrated area ratio (**a**), numbers of tubes formed (**b**) and numbers of endothelial sprouts from aortic rings (**c**) in acetate + PAA-treated group compared to PAA-exposed group (*n* = 5–6). **d**, Schematic diagram of the experimental setting. Aortas were collected from *Clos*-colonized mice for ex vivo aortic EC sprouting assay or isolation of ECs, followed by p16^INK4A^ immunostaining and SA-β-gal staining, in the presence or absence of sodium acetate. **e**,**f**, Immunostaining reveals the expression of proliferative arrest marker p16^INK4A^ (**e**) and SA-β-gal positivity, represented as SA-β-gal^+^ cells (%) (**f**) (*n* = 5–6). **g**, Confocal micrographs of aortic rings represent angiogenic capacity in aortas obtained from *Clos*-colonized mice in response to sodium acetate versus vehicle. Quantitative plot is shown for the number of aortic endothelial sprouts (*n* = 5–6) (right). Scale bars, 50, 100 and 200 μm. Error bars represent s.d. (**a**–**c**,**f**,**g**). Data represent triplicate biologically independent experiments. *P* values were calculated using a two-tailed unpaired Student’s *t*-test. Images created with BioRender.com (**c**,**d**). Source data

To determine whether sodium acetate reverses PAA-induced endothelial dysfunction, we assessed its effect on endothelial angiogenic competence. We demonstrated that sodium acetate enhances the transition of PAA-induced senescent ECs toward an angiogenic phenotype, evidenced by restored migration ability (Fig. 8a) and increased numbers of tubes formed to the magnitude of Veh-PECs (Fig. 8b). Moreover, sodium acetate significantly enhanced endothelial sprouting ex vivo in PAA-exposed aortic rings (Fig. 8c). Notably, sodium acetate increased numbers of sprouted VEC^+^, CD31^+^ ECs, supporting its role in angiogenesis restoration (Supplementary Fig. 6a,b). As a proof of concept, we tested acetate’s capacity in restoring endothelial function in a more complex system, including aortas from *Clos*-colonized mice (Fig. 8d). As expected, our findings mirrored the responses to PAA. Accordingly, sodium acetate significantly reduced senescence hallmarks, p16^INK4a^ and SA-β-gal positivity (Fig. 8e,f), and augmented angiogenic potential, as evidenced by increased numbers of endothelial sprouts (Fig. 8g). These findings suggest that pro-angiogenic effects of acetate supplementation stem from its senomorphic properties, which effectively counteract PAA-induced endothelial senescence and restore endothelial function.

## Discussion

In this study, we confirmed a causal link between gut microbiota and aortic endothelial senescence, identifying *Clostridium* sp. ASF356 and its byproduct PAA as contributors to vascular dysfunction. Our combined metagenomic and metabolomic studies revealed that age-related increases in plasma PAA levels correlate with *ppfor* gene homologs in the aged microbiome. Of note, *Clostridium* sp. ASF356, enriched in aged microbiomes, harbors the *ppfor* gene, facilitating oxidative decarboxylation of phenylpyruvate to PAA, which we confirmed in both in vivo and in vitro models. Functionally, *Clostridium* sp. ASF356 and its metabolite PAA induce endothelial senescence by inducing SASP and epigenetic alterations. Our senolytic D + Q therapy provided a proof of concept for causal contribution of the metaorganismal metabolite to endothelial senescence and dysfunction and angiogenic incompetence. Therefore, abundance of *Clostridium* sp. ASF356 in microbiome or PAA could be introduced as potential key vascular aging biomarkers. Beyond identifying PAA as a pro-senescent metabolite, we also suggest sodium acetate as a potential senomorphic compound that strongly mitigates SASP, restores mitochondrial redox and energy balance, and thereby improves endothelial function. These findings highlight acetate’s senotherapeutic potential for preserving vascular health in aging.

PAA has primarily been recognized as a precursor in the biosynthesis of PAGln in humans^10^ and phenylacetylglycine (PAGly) in rodents^18^. The formation of PAA occurs through both oxidative and nonoxidative phenylalanine metabolism, mediated by PPFOR, VOR and PPDC enzymes. Recent studies have linked PPFOR and PPDC to atherosclerotic CVD^10^. Furthermore, preclinical and clinical studies demonstrated a positive association between PAGln and conditions such as heart failure^11^, thrombotic events^10^ and, notably, cellular senescence^29^. The latter is driven by mitochondrial dysfunction and DNA damage in aged mice. These findings complement our study by supporting the involvement of gut microbiota in driving vascular aging. While only one human study has reported an association between PAA and major adverse cardiovascular events in cardiac patients^30^, the mechanistic implications of PAA remain elusive. Our study establishes a causal role for *Clostridium* sp. ASF356 and its metabolite PAA in driving endothelial senescence and dysfunction, using both mouse and human data. Consistently, our mouse model shows a positive association between *Clostridium* sp. ASF356 and plasma PAA, mirroring findings from TwinsUK cohort, where PAA levels correlated with Clostridium spp. expressing *ppfor* gene. Given the functional conservation of key metabolic pathways between human and mouse microbiomes, we propose that *Clostridium* sp. ASF356 or a functionally equivalent taxon may contribute to vascular aging in humans. Future studies employing genomic insights and targeted culturing will enable us to confirm this hypothesis.

Our findings suggest that *Clostridium* sp. ASF356 regulates circulating PAA levels, thereby promoting aortic stiffening and endothelial senescence and dysfunction. Mechanistically, PAA confers its senescence-promoting effects through excessive NOX4-dependent mitochondrial H_2_O_2_ generation, leading to DNA damage (γ-H2A.X phosphorylation), upregulation of SASP components (IL-1β and IL-6) and proliferative arrest marked by p16^INK4a^, p19^INK4d^ and p21^WAF1/Cip1^ upregulation. PAA-treated ECs also displayed reduced Ki67 expression and impaired viability, consistent with a senescent phenotype. Notably, PAA-driven senescence extended beyond in vitro models, as it also drives SASP and proliferative arrest in aortic ECs in vivo, resembling vascular changes in aged mice. As a proof of concept, senolytic intervention with D + Q could selectively eliminate senescent ECs triggered by the bacterium or its metabolite PAA, validated by reduced proliferative arrest, SASP, DNA damage response (DDR) and lysosomal alterations. The senescence rescue subsequently led to restoration of endothelial function in *Clos*-colonized mice. These findings align with previous studies demonstrating that senolytic D + Q therapy effectively improves vascular phenotype in aged and hypercholesterolemic mice by targeting senescent endothelial and vascular smooth muscle cells^31^. Furthermore, clearance of senescence cells in aortic atheroma lesions has been previously shown to attenuate atherosclerosis progression in chronologically or accelerated (progeroid *Ercc1*^*−/Δ*^) aged mice and athero-prone *Ldlr*-knockout (KO), *3MR* mice fed a high-fat diet^32,33^.

To delve deeper into the mechanistic significance of PAA in endothelial senescence, we employed a chemogenetic approach using recombinant lentiviral DAAO, which selectively generates intracellular H_2_O_2_ in mitochondria. Our results confirmed that mitochondrial H_2_O_2_ induces EC senescence, marked by DNA damage and increased SA-β-gal activity. Aside from this, PAA PAA-induced stress condition counteracts antioxidant defense mechanisms, notably suppressing GPx1, an enzyme essential for H₂O₂ detoxification. In line with previous findings, glutathione peroxidase deficiency promotes p53/p21 pathway activation and endothelial dysfunction, a hallmark of early atherosclerosis^34^. Thus, we believe that PAA may potentially be a pro-oxidant inducer of EC senescence in aging organisms. Beyond oxidative stress, PAA-exposed ECs developed a persistent proinflammatory phenotype, with increased IL-1β, IL-6 and VCAM1 expression. Our experiments by exploiting chemogenetic DAAO system verified that mitochondrial H_2_O_2_ directly upregulates IL-6 and VCAM1, explaining a H_2_O_2_-dependent fashion for PAA-induced SASP activation. These findings suggest PAA as a potent pro-oxidant metabolite that accelerates EC senescence via SASP induction.

To further explore mechanisms underpinning PAA-induced SASP, we relied upon our previous findings, hypothesizing PAA’s role in epigenetic regulation of the SASP components IL-6 and VCAM1 in ECs. We found that PAA-driven H₂O₂ aggravates HDAC4 phosphorylation and cytosolic translocation, leading to VCAM1 de-repression. This aligns with previous reports showing that NOX4-derived H_2_O_2_ modulates the HDAC4 nucleus–cytosol shuttle through both direct oxidation^24^ and H_2_O_2_-regulated phosphorylation^35^. Our data revealed that PAA-mediated H_2_O_2_ indirectly boosts CaMKII phosphorylation of HDAC4, reinforcing its cytosolic accumulation. Furthermore, IL-6 upregulation in PAA-exposed senescent ECs coincided with CaMKII phosphorylation, supporting previous evidence that IL-6/sIL-6R signaling activates CaMKII in a JAK/STAT3-dependent manner in human umbilical vein ECs, thereby regulating endothelial motility and proliferation^36^. Therefore, we believe that PAA both directly (oxidizing by H_2_O_2_) and indirectly (by activating CaMKII) regulates HDAC4 post-translational modification, leading to VCAM1 de-repression in ECs. We also found that epigenetic regulation of VCAM1 disrupts eNOS signaling, ultimately attenuating angiogenesis. As reported previously, eNOS phosphorylation at Ser1177, but not Thr495, by multiple protein kinases can enhance angiogenesis in ischemic tissues^37^. Our findings support the contribution of PAA-induced inflammatory environment to angiogenic incompetence. Mechanistically, PAA-driven oxidative stress and SASP disrupt mitochondrial homeostasis and energy supply, further compromising angiogenic capacity. Previous studies demonstrated that ECs rely on fatty acid β-oxidation to sustain the TCA cycle and OXPHOS during angiogenesis^17^, while also utilizing glycolysis for migration and proliferation. Consistently, our data reveal that PAA inhibits both oxidative phosphorylation and glycolysis, leading to an ATP deficit and impaired angiogenesis. Our findings suggest that an age-related increase in PAA drives metabolic alterations that impair angiogenic capacity through the SASP component VCAM1 in ECs. Given that impaired angiogenesis underlies blood supply crisis in aged patients with arterial stiffness, atherosclerosis and ischemic heart disease^38^, our studies provide a mechanistic basis for PAA as a potential driver of angiogenic incompetence.

To develop a targeted microbiome-based approach against EC senescence, we stand on age-related decline in gut-derived acetate, a key anti-inflammatory SCFA^15^. Given its efficacy in counteracting SASP components (IL-1β and IL-6) in our preliminary studies, we further validated sodium acetate as a potential senomorphic agent in both *Clos*- and PAA-induced EC senescence models. Mechanistically, our findings directed us to the conclusion that acetate drives metabolic reprogramming by activating ACSS2, which converts acetate to acetyl-CoA, fueling the TCA cycle and restoring IDH2 activity, essential for NADH regeneration. This reactivates the ETC and rescues ATP biosynthesis, counteracting PAA-induced metabolic stress. An acetate-driven feedback loop, wherein NADH is rapidly recycled back into NAD^+^, maintains redox homeostasis in these cells. Our supplementary data confirm that acetate-mediated NADH-to-NAD^+^ conversion in PAA-exposed ECs is inhibited by rotenone, a selective complex I inhibitor, underscoring the importance of ETC activity in acetate’s role in restoring energy balance. Consistent with previous studies, sodium acetate effectively rescues EC senescence, compensates for cellular energy homeostasis, necessary for restoring angiogenic capacity. These findings align with previous studies unveiling the instrumental role of acetate in rescuing fatty acid β-oxidation in quiescent ECs^17^ and providing systemic benefits for ameliorating arterial stiffness^39^ and hypertension^40^ in aged mice.

In addition to maintaining energy homeostasis, acetate restores redox balance by attenuating ROS accumulation in PAA-exposed ECs. While mitochondrial OXPHOS may indeed elevate ROS levels, the controlled magnitude and kinetics of H_2_O_2_ production by mitochondrial respiration restricts its capacity to directly induce DNA damage and senescence^41^. Notably, our findings demonstrate that acetate concurrently enhances NAD^+^ availability and antioxidant defense mechanisms by upregulating Nrf2-regulated antioxidant enzymes (Gpx1, Gpx4, Prdx3, Txn1 and Txn2), thereby reinforcing redox homeostasis. This aligns with a recent study by Adeyemi et al., showing that acetate activates Nrf2/HO-1 signaling, leading to GPx, catalase and SOD induction, which protects against doxorubicin-induced cardiac oxidative damage^42^. Similarly, in aged mice, sodium acetate suppressed oxidative stress by upregulating glutathione reductase (GRx), Gpx3 and Prdx1, supporting its vasculoprotective effects both in vivo and in vitro^17^.

A remaining critical question is how acetate modulates Nrf2-driven antioxidant defense to restore mitochondrial redox homeostasis. Our findings indicate that NAD^+^ plays a pivotal role as both a sirtuin deacetylase substrate and a redox-energy regulator. Loss-of-function studies confirm that sodium acetate upregulates NAD^+^-dependent Sirt1, which in turn both deacetylates and activates Nrf2, facilitating its nuclear translocation in ECs. In contrast, PAA promotes the Nrf2 cytosolic shuttle, impairing its antioxidant function. Pharmacological inhibition of Nrf2 by ML385 confirmed that Nrf2 orchestrates the transcription of downstream antioxidant pathways, neutralizing PAA-induced mitochondrial ROS accumulation. Emerging evidence underscores the interplay between Sirt1/Nrf2 signaling and redox-energy regulation. In replicative senescent human umbilical vein ECs, a decline in OXPHOS and glycolytic capacity is linked to Nrf2 suppression^43^. Moreover, Nrf2 repression and disruption of Nrf2/ARE antioxidant pathway contribute to progeroid phenotypes, as seen in Hutchinson–Gilford progeria syndrome^44^, while its upregulation boosts metabolic resilience to oxidative and metabolic stress and reduces atherosclerotic burden^45^. Consistently, long-lived animal models exhibit enhanced Nrf2-regulated antioxidant signaling, suggesting that Nrf2 activation may underpin lifespan extension^46,47^. In agreement with previous studies, our findings prove that Nrf2’s endothelial protection has roots in its upstream regulator, Sirt1. Accordingly, resveratrol, a plant-derived Sirt1 activator, attenuated oxidative stress in coronary arterial ECs and improved vasodilation through Nrf2 activation in mice fed a high-fat diet, whereas its vasculoprotective effects were diminished In Nrf2-deficient mice^48^. A growing number of clinical trials on sirtuin- and NAD^+^-boosting therapies report beneficial effects on arterial stiffening, atherosclerosis and coronary artery disease^49,50^, underscoring the translational potential of Sirt1/Nrf2-targeting interventions. Given these findings, we believe that reduced abundance of acetate-producing bacteria in aging microbiota may disrupt redox-energy balance. Notably, some studies show that germ-free mice, despite lacking acetate-producing bacteria, exhibit extended healthspan^51^, seemingly conflicting with our observations; however, these mice are also devoid of microbial triggers of cellular senescence, such as PAA, PAGln and trimethylamine-*N*-oxide (TMAO), suggesting that the absence of harmful microbial metabolites, rather than acetate depletion, may contribute to their longevity. This highlights the complex interplay between gut microbiota, cellular senescence and host lifespan, reinforcing the need for microbiome-targeted interventions that selectively preserve beneficial microbial metabolites like acetate.

Our studies show that acetate-mediated Sirt1 upregulation not only restores redox and energy balances but also suppresses PAA-induced SASP in ECs. As earlier shown in this work, PAA promotes IL-6-driven VCAM1 signaling, contributing to EC senescence and dysfunction. Our acetate-based intervention identified Sirt1 as a central modulator in reversing these effects. Notably, it has been proposed that Sirt1 deacetylates the RelA/p65 subunit of NF-κB at Lys310, as its direct target, reducing IL-6 expression and attenuating the proinflammatory cascade. These findings align with the targeting aging with metformin (TAME) clinical trial outcomes, where metformin suppresses multiple SASP-associated pathways through Sirt1 activation in diverse cell types such as ECs and adipocytes, extending lifespan and healthspan in humans and mice^52,53^. Conversely, PAA-induced mitochondrial H_2_O_2_ accumulation diminishes Sirt1 expression and its deacetylation activity, thereby sustaining NF-κB-driven inflammatory responses. Previous studies confirm that elevated ROS triggers SASP either directly by NF-κB activation or indirectly by ROS-dependent DDR or telomeric damage^54^. Our collective data support this, demonstrating that acetate mitigates DDR, restores telomere length and enhances hTERT expression, likely through mitochondrial H_2_O_2_ suppression. It may enable PAA-exposed ECs to maintain a high energetic proliferative state, necessary for stimulating angiogenic activity in premature aged ECs^55^. Similarly, resveratrol has been shown to prevent cellular senescence by enhancing telomerase activity in endothelial progenitor cells and vascular smooth muscle cells, via Sirt1-mediated suppression of NF-κB-driven cytokine secretion and upregulation of Nrf2-antioxidant responses in aged nonhuman primates^56,57^. Additionally, our identification of IL-6 as an upstream regulator of CaMKII–VCAM1 signaling highlights acetate’s efficacy in restoring eNOS bioavailability by suppressing the PAA-induced IL-6–VCAM1 axis. Acetate also plays a dual role in replenishing cellular energy and targeting the Sirt1–NF-κB pathway, restoring endothelial sprouting and angiogenic capacity.

One limitation of this study is the absence of direct vascular function assessments like endothelium-dependent vasorelaxation assays. While our ex vivo and in vivo analyses suggest that PAA impacts the vascular aging process, further vascular function and hemodynamic studies are needed to fully characterize PAA-induced endothelial dysfunction. Additionally, while acetate seems to restore endothelial homeostasis, its dose-dependent effects on vascular function require further investigation. Future studies should combine longitudinal microbiome-metabolome profiling with vascular imaging to assess the relevance of *Clostridium* sp. ASF356 and PAA in human vascular aging.

In summary, our findings show that *Clostridium sp. ASF356*, harboring the *ppfor* gene, regulates circulating PAA, a key factor in EC senescence and angiogenic incompetence in aging. We discovered that PAA induces cellular senescence in aortic ECs through H_2_O_2_-regulated SASP. We propose that sodium acetate acts as a senomorphic and redox-homeostatic agent with pro-angiogenic potential to aid recovery from ischemic vascular diseases in older adults. Additionally, genetic engineering of *Clostridium sp. ASF356* to deplete the *ppfor* gene could offer a microbiome-based strategy to decelerate vascular aging and prevent related pathologies; however, further development is needed to target EC senescence and vascular dysfunction in aging populations.

## Methods

### TwinsUK human cohort

#### Plasma metabolomic profiling

To validate the associations between circulating PAA and PAGln concentrations and age, we used the metabolomic data of TwinsUK human aging cohort. The TwinsUK cohort encompasses metabolome data of 1,116 metabolites (including PAA and PAGln) profiled in more than 14,000 adult twins of the same sex between 18 and 95 years old. All participants received no compensation and provided written informed consent. The study was approved by the NRES Committee London-Westminster (REC reference no. EC04/015).

#### Fecal metagenomic profiling

Stool samples were provided by randomly selected twin participants in the TwinsUK cohort, collected at home and stored at −80 °C at St. Thomas’ Hospital for further DNA extraction and library preparation^58^. DNA samples, in multiple batches, were transferred to GenomeScan, Netherlands, for DNA sequencing. Paired-end reads were processed with YAMP (v.0.9.5) using the ‘genomescan’ parameters. Samples with <10 M paired-end reads were discarded to maintain the quality. The taxonomic profiling and quantification of organisms’ relative abundances of all metagenomic samples were quantified using MetaPhlAn v.4.0 (ref. ^59^). The updated species-specific database of markers was built using 99,237 reference genomes representing 16,797 species retrieved from GenBank (January 2019). From this set of reference genomes, we extracted a total of 1,132,166 markers used to profile 13,393 species. This set of species also included 83 species defined by the CAG group approach that were genetically distinct from species represented by isolate genomes and for which the use of unique marker genes limited the potential issues of using metagenomic assemblies reconstructed over multiple samples. Metagenomes were mapped internally in MetaPhlAn v.4.0 against the marker gene database with Bowtie 2 v.2.3.4.3 with the parameter ‘very-sensitive’. The resulting alignments were filtered to remove reads aligned with an MAPQ value < 5, representing an estimated probability of the likelihood of the alignments. The associations between microbial features and metabolites were analyzed using the R package ‘lmerTest’.

### Mouse models

Our research complies with all ethical regulations approved by the Institutional Animal Care and Use Committee at University of Zurich and the Cantonal Ethics Commission Zurich (ZH023/2023 and ZH241/19). Animal care and all experimental protocols were in accordance to the principles of the ‘3Rs’, replacement, reduction and refinement, according to Directive 2010/63/EU of the European Parliament and of the Council of 22 September 2010 on the protection of animals used for scientific purposes.

All mice were housed in controlled environments in Plexiglass cages under a strict 12-h light–dark cycle at an ambient temperature of 23 ± 1 °C and humidity of 55 ± 10%, and fed standard chow diet (including 19% protein, 61% carbohydrate and 7% fat, D11112201, Research Diets) until 3 (as young) or >24 (as aged) months of age. Mice had access to drinking water and food ad libitum.

### Blood and fecal collection and tissue collection

Mice feces were collected and quickly stored at −80 °C for DNA extraction and shotgun metagenomics and quantification of acetate levels.

Mice were killed by CO_2_ inhalation in an area separate from the housing facility. Whole blood was collected by cardiac puncture from mice. Plasma was isolated by centrifugation (125*g*, 8 min, room temperature)^15^. An aliquot of blood samples was incubated for 1 h at room temperature and centrifuged (1,500*g*, 10 min). After blood collection, the heart was perfused for aorta collection.

### Mouse creatinine and BUN assays

Serum creatinine and blood urea nitrogen (BUN) were measured using colorimetric detection kits (Cayman Chemical, 700460 and Invitrogen, EIABUN, respectively), according to the manufacturers’ instructions. Absorbance was read at 495 nm (for creatinine) and 450 nm (for BUN) with a microplate reader.

### Targeted LC–MS/MS analysis of plasma PAA and PAGln levels

PAA and PAGln concentrations in mouse plasma (100 μl) were quantified by liquid chromatography coupled to a high-resolution mass spectrometer (LC–HRMS). Plasma was extracted with frozen ethanol/methanol (−20 °C) containing internal standards (D5-PAA and D5-PAGln, 0.25 µg ml^−1^) and centrifuged (13,000 rpm, 4 °C, 20 min). Supernatants were reconstituted into 100 µl of water:methanol (90:10) for next centrifugation. Supernatants were separated in a Waters HSST3 column (150 × 2.1 mm, 1.8 μm) and analyzed on a Thermo Ultimate 3000 LC system coupled to a Thermo Exploris 120 orbitrap mass spectrometer. Peaks were identified using standards and processed with Tracefinder 3.2 (Thermo Scientific). All MS conditions are detailed in Table 1.

**Table 1:** Plasma PAA and PAGln mass spectrometry conditions

	Polarity	Formula	Adduct	Precursor ion (m/z)	Product ion (m/z)	HCD (%)
PAA	Negative	C_8_H_8_O_2_	-H	135.0452	91.0553	30
PAA-d5	Negative	C_8_H_3_O_2_D_5_	-H	140.0765	96.0869	30
PAGIn	Negative	C_13_H_16_N_2_O_4_	-H	263.1037	127.0513	60
PAGIn-d5	Negative	C_13_H_11_N_2_O_4_D_5_	-H	268.1351	127.0513	60

### Fecal DNA extraction

Fecal genomic DNA was extracted using the FastDNA SPIN kit (MP Biomedicals, 116540600-CF). Samples were homogenized in MT buffer with FastPrep, centrifuged and the supernatant was mixed with PPS buffer. DNA was purified using a column, dissolved in sterile Milli-Q water, and assessed by a NanoDrop 2000 (Thermo Scientific).

### Shotgun metagenomics

Metagenome libraries were generated using a high-quality genomic DNA and libraries were sequenced on an Illumina NovaSeq 6000 PE150 platform (Novogene) with a 250-bp paired-end model.

#### Taxonomic profiling

Taxonomic profiling was performed using kraken2/Bracken2 (parameters, –confidence 0.51)^60^ against the mouse microbiota genome catalog (CMMG) (https://ezmeta.unige.ch/CMMG/Kraken2db/cmmg)^61^. Kraken2 profiles were then subjected to Bracken2 for taxonomic refinement using the corresponding database and read length. Bracken2-corrected CMMG read counts were then normalized for reference genome length and loaded together with available reference genomes taxonomy (https://ezmeta.unige.ch/CMMG/Supplementary_Tables_and_Figures/Tables/Table_S4_curated_taxonomy.tsv) into a phyloseq object for data handling. Only the taxa identified in at least two samples with coverage of at least 100 reads in total were considered for further analyses.

#### Functional profiling

Genomes were screened for presence of K00169 (*vor*) and or K00179 (*ppfor*), the genes encoding the enzymes participated in the phenylalanine metabolism and production of PAA, based on their available KEGG functional annotations (https://ezmeta.unige.ch/CMMG/functional_annotations/mouse/annotations.tsv). The differential abundance of K00169 or K00179 in the identified genome was then added as additional information in the phyloseq object to identify VOR*-* and/or PPFOR-positive taxa in the microbiome of aged and young mice. The association between the abundance of KO and plasma levels of PAA and PAGln were also characterized by a Spearman correlation analysis.

#### Community analysis

Microbiota profiles were characterized using α-diversity and β-diversity^15^. Hill-d0, 1 and 2 indices represent the richness of detected taxa in aged or young microbiome. Pielou’s index (evenness) represents the relative abundance of each taxon in the communities. The influence of age and sex (=cage) was tested on α-diversity and β-diversity metrics using Wilcox univariate nonparametric test and permutational multivariate analysis of variance (PERMANOVA) multivariate test. Age- and sex (=cage)-associated differences in taxa or KO abundance were assessed using the CLR by ANCOM.

### Fecal acetate quantification

Fecal pellets were homogenized, and supernatants were analyzed using a LaChrom HPLC system (Merck-Hitachi) equipped with a SecurityGuard Carbo-H cartridge (4 × 3.0 mm, Phenomenex) and a Rezex ROA-Organic Acid H^+^ column (300 × 7.8 mm, Phenomenex). Separation was performed with a refractive index detector (L-2490, Merck-Hitachi) for analyte quantification. Data were processed using EZChrom A.04.10 (Agilent).

### In vitro screening of PAA and PAGln formation by *Clostridium* sp. ASF356

Axenic culture of *Clostridium* sp. ASF356, a strain from the Altered Schaedler Flora, was obtained from M. Wannemuehler at the Iowa State University. *Clostridium* sp. ASF356 was reactivated by a 1% (v/v) inoculation in either brain heart infusion or yeast casitone fatty acids medium anaerobically at 37 °C. Overnight saturated pre-cultures were transferred (1%, v/v) into the fresh medium and cells were grown for 46 h at 37 °C. Bacterial culture supernatants were quantified using LC–MS/MS system. The raw data were processed using Tracefinder 3.2 (Thermo Scientific).

### Mouse treatments

#### In vivo mouse bacterial colonization

The 10–12-week-old, C57BL/6 male mice were administered an antibiotic cocktail (containing 0.5 mg ml^−1^ ampicillin, 0.5 mg ml^−1^ neomycin, 0.25 mg ml^−1^ vancomycin and 0.5 mg ml^−1^ metronidazole) in drinking water for a total of 2 weeks. After 2 days, mice were administered 0.2 ml *Clostridium* sp. ASF356 bacterial suspension by oral gavage inside a biological safety cabinet. For robust colonization, oral gavages were repeated for 3 consecutive days, followed by every 7 days for 4 weeks. Mice were maintained on a sterilized standard chow diet (containing phenylalanine 4.15 g per 100 g; Research Diets) until they were killed for blood collection and tissue collection. To verify the successful colonization, DNA was isolated from fecal content obtained from the distal colon and used for a PCR reaction.

#### In vivo senolytic therapy of colonized mice

Mice colonized with *Clostridium* sp. *ASF356* for 4 weeks, followed by treatment with a senolytic cocktail consisting of dasatinib (5 mg kg^−1^, Sigma-Aldrich, CDS023389) and quercetin (50 mg kg^−1^, Sigma-Aldrich, Q4951) by oral gavage daily for 3 consecutive days, followed by a 7-day resting period. Mice were maintained on a sterilized standard chow diet (containing phenylalanine 4.15 g per 100 g; Research Diets) until they were killed for blood and aorta collection.

#### In vivo PAA administration

Mice were administered PAA (50 mg kg^−1^ d^−1^, Sigma-Aldrich, P16621) via intraperitoneal (i.p.) injection daily for 4 weeks. Mice were killed for blood and aorta collection.

### Cell line

Primary human aortic ECs (HAECs; Lonza, CC-2535) were cultured in EBM-2 medium (Lonza, 00190860) supplemented with EGM-2 (10% FBS, 2 mM l-glutamine, 100 μg ml^−1^ penicillin–streptomycin, Lonza, CC-3162/6) at 37 °C, 5% CO_2_. Cells at passages 4–5 (proliferating) and 15–17 (replicative senescence) were studied. Cellular senescence was confirmed by proliferative arrest (reduced cell count), SA-β-gal staining and p21^WAF1/Cip1^ immunoblots.

### Mouse aortic endothelial cell isolation

Thoracic aortas were excised from mice, cleaned in ice-cold Dulbecco’s phosphate-buffered saline (Gibco, 14190144), and digested with collagenase type II (1 mg ml^−1^, 37 °C, 45 min; Sigma-Aldrich, C2–28). The cell suspension was filtered (70 µm), centrifuged (300*g*, 10 min) and resuspended in EBM-2 medium with EGM-2 supplements (37 °C, 5% CO_2_). Cells (passages 2–3) were used for senescence studies.

### Endothelial cell senescence models

#### Replicative senescence

HAECs (p.4–5) seeded in EGM-2 (5,000 cells per cm^2^) were passaged every 48 h until the proliferation is suppressed and replicative senescence phenotype is confirmed (SEC; p.15–17). Cellular senescence was verified by SA-β-gal staining and marked reduction in cell count (stained with Trypan blue)^62^. In parallel, another series of cells (p.4–5) cultured in a growth medium for 48 h was identified as a proliferative control (PEC).

#### H_2_O_2_-induced premature senescence

To induce premature senescence, PECs (p.4–5) were cultured in EGM-2 (+10% FBS) and treated with exogenous H_2_O_2_ (50 μM) for 4 h. The culture medium was then replaced and cells were maintained for up to 72 h.

#### Senescence-associated-β-galactosidase assay

Cultured HAECs were stained for SA-β-galactosidase according to the manufacturer’s instructions (Merck Millipore, KAA002). In brief, cells were fixed and stained with x-gal solution overnight at 37 °C. Senescent cells, illuminated as blue-stained cells, were captured under a Leica TCS-SP8 confocal microscope and analyzed using ImageJ software 1.54j (Fiji).

### CellRox Green staining

Cells were grown to confluence in growth medium and treated with CellROX Green reagent at a final concentration of 5 μM (Invitrogen, C10444) for 30 min at 37 °C to detect intracellular ROS by a Leica TCS-SP8 microscope, visualized by LAS-X-Office 1.4.6.28433. The fluorescence intensity was evaluated using ImageJ 1.54j.

### Lentiviral DAAO-HyPer7.2 transduction

HAECs were transduced with lentivirus-DAAO-HyPer7.2 targeted to cell mitochondria (multiplicity of infection 20–50) in serum-free medium, replaced with 10% FBS medium after 5 h. After 48–72 h, cells were treated with l-alanine (control) or d-alanine (10 mM) to generate mitochondrial H_2_O_2_ and subsequently subjected to senescence and signaling studies.

### HyPer7.2 fluorescence imaging

HAECs were transduced with adenovirus serotype 5 (AV5)-HyPer7.2-mito (multiplicity of infection 1,000)^25^. Cells were treated with exogenous H_2_O_2_ or PAA, washed and incubated in HEPES-buffered solution (pH 7.4) for 2 h at 37 °C. Coverslips were mounted on a live-cell imaging platform for real-time fluorescence imaging. The ratiometric HyPer7.2 biosensor was excited at 420 nm and 490 nm, with emission recorded at 530 nm using Metafluor Software (Molecular Devices)^63–65^. Mitochondrial H_2_O_2_ was quantified as the R/R_0_ ratio (490/420 nm signals).

### Cellular acetyl-CoA measurement

Cultured HAECs were washed, scratched and centrifuged (500*g*, 5 min, 4 °C). Cytosolic extracts were precipitated with perchloric acid, neutralized (6.0 < pH < 8.0) and analyzed for acetyl-CoA levels using an Acetyl-CoA Assay kit (Sigma-Aldrich, MAK039).

### Relative NAD^+^ measurement

Cultured HAECs were washed, suspended in NADH/NAD extraction buffer (Sigma-Aldrich, MAK037), and centrifuged (13,000 rpm, 4 °C, 5 min). Supernatant was split for total NAD (NAD_t_) and NADH measurement. Absorbance was measured at 450 nm and NAD⁺ levels were calculated as ‘NAD_t_ – NADH’.

### Real-time quantitative PCR

Total RNA from cells and aortas was extracted using TRIzol Reagent (Sigma-Aldrich, T9424), according to the standard protocol^64–66^, for real-time PCR using Power SYBR Green PCR Master Mix (Thermo Fisher Scientific, 4367659). All the primer sequences are listed in Supplementary Table 1.

### siRNA transfection

Cells were grown to 70–80% confluence for transfection with specific siRNAs (listed in Supplementary Table 2) using Lipofectamine RNAiMAX transfection reagent (Invitrogen, 13778150). The silencing efficiency was confirmed by immunoblots.

### Immunoblotting

Aortic tissues and ECs were lysed in ice-cold RIPA buffer (Thermo Fisher Scientific, 89901) or nuclear and cytoplasmic extraction reagents (Thermo Fisher Scientific, 78835) supplemented with Halt protease and phosphatase inhibitors cocktail (Thermo Fisher Scientific, 78442). Total protein was quantitated using the BCA protein assay kit (Thermo Fisher Scientific, 23225). Equal amounts of proteins (20 µg) were separated in 10% SDS–PAGE gel, followed by electroteranfer and further incubation with specific antibodies. The protein bands were visualized using Amersham Imager 600 (GE Healthcare) and quantitative densitometric analyses were performed using ImageJ 1.54j.

All antibodies used in immunoblotting are listed in Supplementary Table 3.

### Opal Multiplex immunofluorescence

Murine aortas and ECs were formalin-fixed and paraffin-embedded. Then, 4-μm tissue sections were stained with primary antibodies (1:100 dilution) using the Opal multiplex immunostaining system (Akoya Biosciences). Staining conditions were optimized using chromogenic DAB detection (Leica Biosystems). Multiplex immunostaining for p-HDAC4^Ser632^, CD31, p16^INK4a^, VCAM1, γ-H2A.X^Ser139^ and 4,6-diamidino-2-phenylindole (DAPI) was performed using the Opal 7-Color Automation immunohistochemistry kit on a Leica Bond Rx autostainer. Antibody/fluorophore pairs were spectrally colocalized as per manufacturer recommendations. Slides were scanned using the Vectra Polaris Imaging System, visualized by Phenochart 1.2.0 (Akoya Biosciences).

### Seahorse Mito Stress assay

Cells were seeded at 5,000 cells per well in Seahorse XF24 plates (Agilent Technologies). On the experiment day, cells were washed and incubated in culture medium supplemented with 25 mM glucose, 2 mM glutamine and 1 mM pyruvate (pH 7.4). For a standard mitochondrial stress test, oligomycin (1 μM), FCCP (3 μM) and antimycin A/rotenone (0.5 μM each) were sequentially injected to assess oxygen consumption rate (OCR) and ECAR over a 3-min period. At any condition, five consecutive OCR/ECAR measurements were recorded using the Seahorse XFe96 Analyzer (Agilent Technologies). Data were normalized to total protein per well.

### Endothelial cell migration assay

Cells were seeded 24 h before treatment and grown to confluence. A cell-free gap was created using a sterile 200-μl pipette tip, and monolayer migration was monitored^62^. Phase-contrast live-cell imaging (Olympus IX81, Hamamatsu C11440) captured images every 15 min from time point *t* = 0 to *t* = 16 h at ×6.4 magnification in a humidified incubator (37 °C, 5% CO_2_). The migrated area ratio at 16 h was quantified using ImageJ 1.54j.

### Endothelial tube formation assay

The angiogenic capacity of ECs was assessed by tube formation assay onto Matrigel (Corning 356234) at a subconfluent level^62^. Images were recorded every 15 min for 16 h at ×6.4 magnification using a phase-contrast Olympus IX81 microscope (Hamamatsu C11440 detector, 1 megapixel, 16 bit). Tube formation was analyzed using ImageJ Angiogenesis Analyzer (v.1.54j).

### Ex vivo aortic ring sprouting assay

Aortic ring sprouting was assessed by culturing the fragments of murine aortas (2-mm) onto Matrigel and subsequently imaged daily (for 5 days) using an incubator-equipped phase-contrast Olympus IX81 microscope with the Hamamatsu (C11440) detector, followed by the analysis of the number of aortic sprouts^62^.

### Aortic sprout immunofluorescence staining

Murine aortic endothelial sprouts on Matrigel were fixed in 4% PFA, permeabilized with 0.25% Triton X-100 and blocked, followed by incubation with primary and secondary antibodies (1:50 to 1:100). Confocal imaging (Leica TCS-SP8) was performed, and nuclei were labeled using DAPI-containing mounting medium for 30 min.

### Ex vivo endothelium-dependent relaxation assay

Murine aortas (2 mm) were mounted in a 5-ml organ chamber filled with Krebs-Ringer bicarbonate solution (37 °C, pH 7.4, bubbled with 95% O_2_/5% CO_2_) and connected to an isometric force transducer (PowerLab 8/30 and LabChart v.7.2.5, AD Instruments) for continuous isometric tension recording (Multi-Myograph 610M, Danish Myo Technology). Concentration-response curves were obtained in responses to increasing concentrations of acetylcholine (Ach, 10^−9^ to 10^−5^ M; Sigma-Aldrich, A6625)^66^.

### Statistics and reproducibility

No statistical methods were used to predetermine sample sizes, but similar sample sizes were used in previous publications^15,62,66^. In TwinsUK human study, participants that exhibited values below the detection level (0) for PAA or PAGln were considered as not available. Accordingly, PAA and PAGln were measured in plasma samples of 7,303 individuals by Metabolon using a nontargeted UPLC–MS/MS platform. No animals or data points were excluded from experiments or analyses. No method of randomization was used to assign animals to experimental groups. Animals were monitored daily for sickness symptoms and body weight during their lifespan. They were killed immediately at the clinical end point when recommended by veterinary and biological service staff members. The investigators in this study were blinded to the conditions of the experiments, except for oral gavage (for *Clos*, PAA or D + Q administration) to avoid cross-contamination within the groups. *n* represents the number of samples or animals used. At least three independent triplicated experiments were performed for each experimental setup. Data are expressed as mean ± s.d. or s.e.m. and *P* < 0.05 was defined as statistically significant.

Statistical analysis was performed using GraphPad Prism 9 (v.9.5.1), R (v.1.26.1) and R package ‘lmerTest’ (v.3.1.3, function ‘lmer’). Comparisons between two groups were conducted using unpaired two-tailed Student’s *t*-test, two-tailed Mann–Whitney *U*-test and one-way or two-way analysis of variance (ANOVA) with Tukey’s post hoc test. Heatmaps were generated using the vegan v.2.5-5 package (https://Github.com/vegan). Spearman rank correlation was used to analyze associations between plasma levels of metabolites and microbial taxa. Multiple group comparisons were tested using Kruskal–Wallis and PERMANOVA was used for analysis of α- and β-diversity. Multiple comparisons and associated *P* values were false discovery rate corrected. Data distribution was assumed to be normal but this was not formally tested.

### Reporting summary

Further information on research design is available in the Nature Portfolio Reporting Summary linked to this article.

## Supplementary information

Supplementary Information Supplementary Figs. 1–10 and Supplementary Tables 1–3.

Reporting Summary

Supplementary Data 1 Supplementary Fig. 1.

Supplementary Data 2 Supplementary Fig. 2.

Supplementary Data 3 Supplementary Fig. 3.

Supplementary Data 4 Supplementary Fig. 4.

Supplementary Data 5 Supplementary Fig. 9.

## Source data

Source Data Fig. 1 Statistical Source Data, LC–MS/MS readouts, unprocessed western blot membranes.

Source Data Fig. 2 Statistical Source Data, unprocessed western blot membranes.

Source Data Fig. 3 Statistical Source Data, unprocessed western blot membranes.

Source Data Fig. 4 Statistical Source Data, unprocessed western blot membranes.

Source Data Fig. 5 Statistical Source Data, unprocessed western blot membranes.

Source Data Fig. 6 Statistical Source Data.

Source Data Fig. 7 Statistical Source Data, unprocessed western blot membranes.

Source Data Fig. 8 Statistical Source Data.

Source Data Extended Data Fig. 1 Statistical Source Data.

Source Data Extended Data Fig. 2 Statistical Source Data.

Source Data Extended Data Fig. 3 Statistical Source Data, unprocessed western blot membranes.

Source Data Extended Data Fig. 4 Statistical Source Data, unprocessed western blot membranes.

Source Data Extended Data Fig. 5 Statistical Source Data, unprocessed western blot membranes.

Source Data Extended Data Fig. 6 Statistical Source Data, unprocessed western blot membranes.

Source Data Extended Data Fig. 7 Statistical Source Data, unprocessed western blot membranes.

Source Data Extended Data Fig. 8 Statistical Source Data, unprocessed western blot membranes.

Source Data Extended Data Fig. 9 Statistical Source Data, unprocessed western blot membranes.

Source Data Extended Data Fig. 10 Statistical Source Data, unprocessed western blot membranes.

## Data Availability

All data from shotgun metagenomics analyses in this study have been deposited in the National Center for Biotechnology Information Sequence Read Archive under accession no. PRJNA1242241. Screening of K00169 and K00179 was conducted using KEGG annotations, which are accessible at https://ezmeta.unige.ch/CMMG/functional_annotations/mouse/annotations.tsv. The targeted metabolomics data, including Thermo’s raw data files and processed datasets analyzed by Tracefinder 3.2, have been securely stored on an institutional research repository at University of Lausanne. These data are available upon request from the corresponding authors and subject to institutional data-sharing policies. The human study data used in this research are maintained by the Department of Twin Research at King’s College London and are not publicly available due to patient privacy and data protection regulations. Researchers seeking access must apply through the established procedures outlined by the Wellcome Trust, following the guidelines provided at https://twinsuk.ac.uk/researchers/explore-our-data-and-samples/. Access is granted upon approval and may require specific ethical and governance clearances. All other data and reagents that support the findings of this study are available upon reasonable request from the corresponding authors. Source Data are provided with this paper.

## References

[1] Bloom, S. I. et al. Mechanisms and consequences of endothelial cell senescence. *Nat. Rev. Cardiol.* **20**, 38–51 (2023). 35853997 10.1038/s41569-022-00739-0 PMC10026597

[2] Yrrell, D. J. & Goldstein, D. R. Ageing and atherosclerosis: vascular intrinsic and extrinsic factors and potential role of IL-6. *Nat. Rev. Cardiol.* **18**, 58–68 (2021). 32918047 10.1038/s41569-020-0431-7 PMC7484613

[3] Donato, A. J. et al. Mechanisms of dysfunction in the aging vasculature and role in age-related disease. *Circ. Res.* **123**, 825–848 (2018). 30355078 10.1161/CIRCRESAHA.118.312563 PMC6207260

[4] Han, Y. & Kim, S. Y. Endothelial senescence in vascular diseases: current understanding and future opportunities in senotherapeutics. *Exp. Mol. Med.* **55**, 1–12 (2023). 36599934 10.1038/s12276-022-00906-w PMC9898542

[5] Saeedi Saravi, S. S. & Feinberg, M. W. Can removal of zombie cells revitalize the aging cardiovascular system? *Eur. Heart J.* **45**, 867–869 (2024). 38190315 10.1093/eurheartj/ehad849

[6] Allemann, M.S. et al. Targeting redox system for cardiovascular regeneration in aging. *Aging Cell* **22**, e14020 (2023). 37957823 10.1111/acel.14020 PMC10726899

[7] Witkowski, M. et al. Gut microbiota and cardiovascular disease. *Circ. Res.* **127**, 553–570 (2020). 32762536 10.1161/CIRCRESAHA.120.316242 PMC7416843

[8] Fromentin, S. et al. Microbiome and metabolome features of the cardiometabolic disease spectrum. *Nat. Med.* **28**, 303–314 (2022). 35177860 10.1038/s41591-022-01688-4 PMC8863577

[9] Chakaroun, R. M. et al. The potential of tailoring the gut microbiome to prevent and treat cardiometabolic disease. *Nat. Rev. Cardiol.* **20**, 217–235 (2023). 36241728 10.1038/s41569-022-00771-0

[10] Zhu, Y. et al. Two distinct gut microbial pathways contribute to meta-organismal production of phenylacetylglutamine with links to cardiovascular disease. *Cell Host. Microbe* **31**, 18–32 (2023). 36549300 10.1016/j.chom.2022.11.015 PMC9839529

[11] Poesen, R. et al. Microbiota-derived phenylacetylglutamine associates with overall mortality and cardiovascular disease in patients with CKD. *J. Am. Soc. Nephrol.* **27**, 3479–3487 (2016). 27230658 10.1681/ASN.2015121302 PMC5084895

[12] Romano, K. A. et al. Gut microbiota-generated phenylacetylglutamine and heart failure. *Circ. Heart Fail.* **16**, e009972 (2023). 36524472 10.1161/CIRCHEARTFAILURE.122.009972 PMC9851997

[13] Morita, M. et al. Phenylacetic acid stimulates reactive oxygen species generation and tumor necrosis factor-α secretion in vascular endothelial cells. *Ther. Apher. Dial.* **15**, 147–150 (2011). 21426506 10.1111/j.1744-9987.2010.00887.x

[14] van der Hee, B. & Wells, J. M. Microbial regulation of host physiology by short-chain fatty acids. *Trends Microbiol.* **29**, 700–712 (2021). 33674141 10.1016/j.tim.2021.02.001

[15] Saeedi Saravi, S. S. et al. Dietary omega-3 fatty acid suppresses age-associated thrombotic potential via gut microbiota modulation. *iScience.* **24**, 102897 (2021). 34401676 10.1016/j.isci.2021.102897 PMC8355916

[16] Marques, F. Z. et al. High-fiber diet and acetate supplementation change the gut microbiota and prevent the development of hypertension and heart failure in hypertensive mice. *Circulation* **135**, 964–977 (2017). 27927713 10.1161/CIRCULATIONAHA.116.024545

[17] Kalucka, J. et al. Quiescent endothelial cells upregulate fatty acid β-oxidation for vasculoprotection via redox homeostasis. *Cell Metab.* **28**, 881–894 (2018). 30146488 10.1016/j.cmet.2018.07.016

[18] Nemet, I. et al. A cardiovascular disease-linked gut microbial metabolite acts via adrenergic receptors. *Cell.* **180**, 862–877 (2020). 32142679 10.1016/j.cell.2020.02.016 PMC7402401

[19] Luo, Y. et al. Predicted visceral adiposity index in relation to risk of coronary heart disease and all-cause mortality: insights from NHANES. *Front. Endocrinol.* **14**, 1296398 (2024). 10.3389/fendo.2023.1296398 PMC10801171 38260165

[20] Wiley, C. D. & Campisi, J. The metabolic roots of senescence: mechanisms and opportunities for intervention. *Nat. Metab.* **3**, 1290–1301 (2021). 34663974 10.1038/s42255-021-00483-8 PMC8889622

[21] Wu, Y. et al. Phosphoglycerate dehydrogenase activates PKM2 to phosphorylate histone H3T11 and attenuate cellular senescence. *Nat. Commun.* **14**, 1323 (2023). 36899022 10.1038/s41467-023-37094-8 PMC10006232

[22] Kabacik, S. et al. The relationship between epigenetic age and the hallmarks of aging in human cells. *Nat. Aging*. **2**, 484–493 (2022). 37034474 10.1038/s43587-022-00220-0 PMC10077971

[23] Lee, Y. et al. Histone deacetylase 4 reverses cellular senescence via DDIT4 in dermal fibroblasts. *Aging.* **14**, 4653–4672 (2022). 35680564 10.18632/aging.204118 PMC9217707

[24] Schader, T. et al. Oxidation of HDAC4 by Nox4-derived H_2_O_2_ maintains tube formation by endothelial cells. *Redox Biol*. **36**, 101669 (2020). 32818796 10.1016/j.redox.2020.101669 PMC7452117

[25] Saeedi Saravi, S. S. et al. Differential endothelial signaling responses elicited by chemogenetic H_2_O_2_ synthesis. *Redox Biol.* **36**, 101605 (2020). 32590330 10.1016/j.redox.2020.101605 PMC7322171

[26] Yang, D. et al. HDAC4 regulates vascular inflammation via activation of autophagy. *Cardiovasc. Res.* **114**, 1016–1028 (2018). 29529137 10.1093/cvr/cvy051

[27] Mann, E. R. et al. Short-chain fatty acids: linking diet, the microbiome and immunity. *Nat. Rev. Immunol*. **24**, 577–595 (2024). 38565643 10.1038/s41577-024-01014-8

[28] Das, A. et al. Impairment of an endothelial NAD^+^-H_2_S signaling network is a reversible cause of vascular aging. *Cell* **173**, 74–89.e20 (2018). 29570999 10.1016/j.cell.2018.02.008 PMC5884172

[29] Yang, H. et al. Gut microbial-derived phenylacetylglutamine accelerates host cellular senescence. *Nat. Aging.* **5**, 401–418 (2025). 39794469 10.1038/s43587-024-00795-w

[30] Nemet, I. et al. Atlas of gut microbe-derived products from aromatic amino acids and risk of cardiovascular morbidity and mortality. *Eur. Heart J.* **44**, 3085–3096 (2023). 37342006 10.1093/eurheartj/ehad333 PMC10481777

[31] Roos, C. M. et al. Chronic senolytic treatment alleviates established vasomotor dysfunction in aged or atherosclerotic mice. *Aging Cell* **15**, 973–977 (2016). 26864908 10.1111/acel.12458 PMC5013022

[32] Childs, B. G. et al. Senescent intimal foam cells are deleterious at all stages of atherosclerosis. *Science* **354**, 472–477 (2016). 27789842 10.1126/science.aaf6659 PMC5112585

[33] Zhu, Y. et al. The Achilles’ heel of senescent cells: from transcriptome to senolytic drugs. *Aging Cell* **14**, 644–658 (2015). 25754370 10.1111/acel.12344 PMC4531078

[34] Jiang, H. et al. Mechanisms of oxidized LDL-mediated endothelial dysfunction and its consequences for the development of atherosclerosis. *Front. Cardiovasc. Med.* **9**, 925923 (2022). 35722128 10.3389/fcvm.2022.925923 PMC9199460

[35] Matsushima, S. et al. Increased oxidative stress in the nucleus caused by Nox4 mediates oxidation of HDAC4 and cardiac hypertrophy. *Circ. Res.* **112**, 651–663 (2013). 23271793 10.1161/CIRCRESAHA.112.279760 PMC3574183

[36] O’Brien, B. J. et al. CaMKIIδ is upregulated by pro-inflammatory cytokine IL-6 in a JAK/STAT3-dependent manner to promote angiogenesis. *FASEB J.* **35**, e21437 (2021). 33749880 10.1096/fj.202002755R PMC8223739

[37] Fukumura, D. et al. Predominant role of endothelial nitric oxide synthase in vascular endothelial growth factor-induced angiogenesis and vascular permeability. *Proc. Natl Acad. Sci. USA.* **98**, 2604–2609 (2001). 11226286 10.1073/pnas.041359198 PMC30185

[38] Chen, X. et al. Therapeutic angiogenesis and tissue revascularization in ischemic vascular disease. *J. Biol. Eng.* **17**, 13 (2023). 36797776 10.1186/s13036-023-00330-2 PMC9936669

[39] Lindquist, A. et al. Oral supplementation with the short-chain fatty acid acetate ameliorates age-related aortic stiffening in mice. *Physiology* **38**, S1.5732615 (2023). 10.59368/agingbio.20240033 PMC11785404 39897133

[40] Poll, B. G. et al. Acetate, a short-chain fatty acid, acutely lowers heart rate and cardiac contractility along with blood pressure. *J. Pharmacol. Exp. Toxicol.* **377**, 39–50 (2021). 10.1124/jpet.120.000187 PMC7985618 33414131

[41] van Soest, D. M. K. et al. Mitochondrial H_2_O_2_ release does not directly cause damage to chromosomal DNA. *Nat. Commun.* **15**, 2725 (2024). 38548751 10.1038/s41467-024-47008-x PMC10978998

[42] Adeyemi, D. H. et al. Sodium acetate ameliorates doxorubicin-induced cardiac injury via upregulation of Nrf2/HO-1 signaling and downregulation of NFκB-mediated apoptotic signaling in Wistar rats. *Naunyn Schmiedebergs Arch. Pharmacol.* **397**, 423–435 (2024). 37458777 10.1007/s00210-023-02620-4

[43] Kuosmanen, S. M. et al. MicroRNAs mediate the senescence-associated decline of NRF2 in endothelial cells. *Redox Biol*. **18**, 77–83 (2018). 29986211 10.1016/j.redox.2018.06.007 PMC6037909

[44] Kubben, N. et al. Repression of the antioxidant NRF2 pathway in premature aging. *Cell* **165**, 1361–1374 (2016). 27259148 10.1016/j.cell.2016.05.017 PMC4893198

[45] Li, J. et al. Targeting the Nrf2 pathway against cardiovascular disease. *Expert Opin. Ther. Targets* **13**, 785–794 (2009). 19530984 10.1517/14728220903025762

[46] Lewis, K. N. et al. Regulation of Nrf2 signaling and longevity in naturally long-lived rodents. *Proc. Natl Acad. Sci. USA* **112**, 3722–3727 (2015). 25775529 10.1073/pnas.1417566112 PMC4378420

[47] Sykiotis, G. P. et al. The role of the antioxidant and longevity-promoting Nrf2 pathway in metabolic regulation. *Curr. Opin. Clin. Nutr. Metab. Care* **14**, 41–48 (2011). 21102319 10.1097/MCO.0b013e32834136f2 PMC3092636

[48] Ungavri, Z. et al. Resveratrol confers endothelial protection via activation of the antioxidant transcription factor Nrf2. *Am. J. Physiol. Heart Circ. Physiol.* **299**, H18–H24 (2010). 20418481 10.1152/ajpheart.00260.2010 PMC2904129

[49] Kane, A. E. & Sinclair, D. A. Sirtuins and NAD^+^ in the development and treatment of metabolic and cardiovascular diseases. *Circ. Res.* **123**, 868–885 (2018). 30355082 10.1161/CIRCRESAHA.118.312498 PMC6206880

[50] Martens, C. R. et al. Chronic nicotinamide riboside supplementation is well-tolerated and elevates NAD+ in healthy middle-aged and older adults. *Nat. Commun.* **9**, 1286 (2018). 29599478 10.1038/s41467-018-03421-7 PMC5876407

[51] Thevaranjan, N. et al. Age-associated microbial dysbiosis promotes intestinal permeability, systemic inflammation, and macrophage dysfunction. *Cell Host Microbe* **21**, 455–466 (2017). 28407483 10.1016/j.chom.2017.03.002 PMC5392495

[52] Kulkarni, A. S. et al. Benefits of metformin in attenuating the hallmarks of aging. *Cell Metab.* **32**, 15–30 (2020). 32333835 10.1016/j.cmet.2020.04.001 PMC7347426

[53] Le Pelletier, L. et al. Metformin alleviates stress-induced cellular senescence of aging human adipose stromal cells and the ensuing adipocyte dysfunction. *eLife* **10**, e62635 (2021). 34544550 10.7554/eLife.62635 PMC8526089

[54] Grootaert, M. O. J. Cell senescence in cardiometabolic diseases. *NPJ Aging* **10**, 46 (2024). 39433786 10.1038/s41514-024-00170-4 PMC11493982

[55] Moslehi, J. et al. Telomeres and mitochondria in the aging heart. *Circ. Res.* **110**, 1226–1237 (2012). 22539756 10.1161/CIRCRESAHA.111.246868 PMC3718635

[56] Xia, L. et al. Resveratrol reduces endothelial progenitor cells senescence through augmentation of telomerase activity by Akt-dependent mechanisms. *Br. J. Pharmacol.* **155**, 387–394 (2008). 18587418 10.1038/bjp.2008.272 PMC2567879

[57] Csiszar, A. et al. Age-associated proinflammatory secretory phenotype in vascular smooth muscle cells from the non-human primate *Macaca mulatta*: reversal by resveratrol treatment. *J. Gerontol. A Biol. Sci. Med. Sci.* **67**, 811–820 (2012). 22219513 10.1093/gerona/glr228 PMC3536544

[58] Wood, D. E. et al. Improved metagenomic analysis with Kraken 2. *Genome Biol.* **20**, 257 (2019). 31779668 10.1186/s13059-019-1891-0 PMC6883579

[59] Keiser, S. et al. Comprehensive mouse microbiota genome catalog reveals major difference to its human counterpart. *PLoS Comput. Biol.* **18**, e1009947 (2022). 35259160 10.1371/journal.pcbi.1009947 PMC8932566

[60] Visconti, A. et al. Interplay between the human gut microbiome and host metabolism. *Nat. Commun.* **10**, 4505 (2019). 31582752 10.1038/s41467-019-12476-z PMC6776654

[61] Blanco-Míguez, A. et al. Extending and improving metagenomic taxonomic profiling with uncharacterized species using MetaPhlAn 4. *Nat. Biotechnol.* **41**, 1633–1644 (2023). 36823356 10.1038/s41587-023-01688-w PMC10635831

[62] Shabanian, K. et al. AQP1 differentially orchestrates endothelial cell senescence. *Redox Biol.* **76**, 103317 (2024). 39180980 10.1016/j.redox.2024.103317 PMC11388013

[63] Eroglu, E. et al. Discordance between eNOS phosphorylation and activation revealed by multispectral imaging and chemogenetic methods. *Proc. Natl Acad. Sci. USA* **116**, 20210–20217 (2019). 31527268 10.1073/pnas.1910942116 PMC6778257

[64] Spyropoulos, F. et al. Metabolomic and transcriptomic signatures of chemogenetic heart failure. *Am. J. Physiol. Heart Circ. Physiol.* **322**, H451–H465 (2021). 10.1152/ajpheart.00628.2021 PMC8896991 35089810

[65] Sorrentino, A. et al. Reversal of heart failure in a chemogenetic model of persistent cardiac redox stress. *Am. J. Physiol. Heart Circ. Physiol.* **317**, H617–H626 (2019). 31298558 10.1152/ajpheart.00177.2019 PMC6766720

[66] Saeedi Saravi, S. S. et al. Long-term dietary n3 fatty acid prevents aging-related cardiac diastolic and vascular dysfunction. *Vasc. Pharmacol.* **150**, 107175 (2023). 10.1016/j.vph.2023.107175 37105373

