# Supplementary material for: Gut microbiota-dependent increase in phenylacetic acid induces endothelial cell senescence during aging
Source: Nat Aging. 2025 May 12;5(6):1025–45. doi: 10.1038/s43587-025-00864-8 (PMC12176623; doi:10.1038/s43587-025-00864-8)
Supplement: Supplementary file 1 — Supplementary Figs. 1–10 and Supplementary Tables 1–3. [file 43587_2025_864_MOESM1_ESM.pdf]

nature aging

Supplementary information

https://doi.org/10.1038/s43587-025-00864-8

Gut microbiota-dependent increase in phenylacetic acid induces endothelial cell senescence during aging

In the format provided by the authors and unedited

Supplementary Information

Supplementary Figures

Supplementary Figure 1

Supplementary Figure 2

Supplementary Figure 3

Supplementary Figure 4

Supplementary Figure 5

Supplementary Figure 6

Supplementary Figure 7

Supplementary Figure 8

Supplementary Figure 9

Supplementary Figure 10

Supplementary Tables

Supplementary Table 1

Supplementary Table 2

Supplementary Table 3

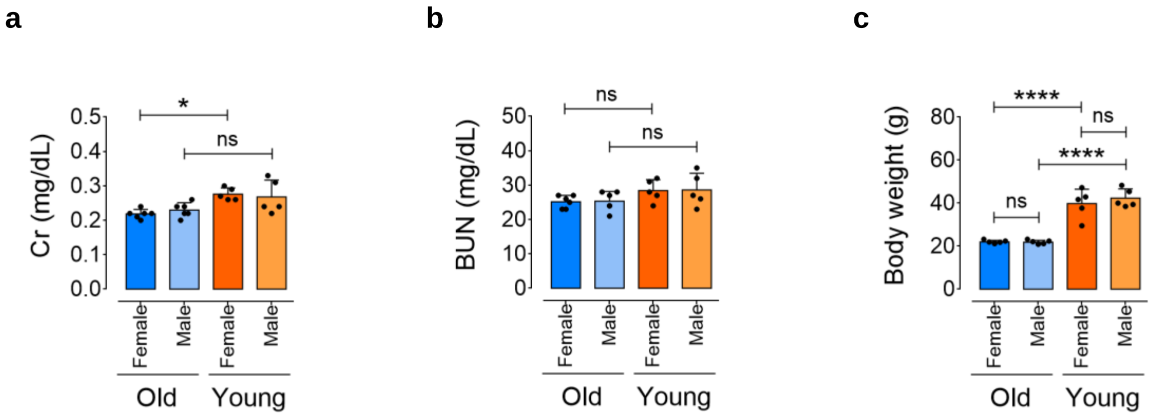

Supplementary Fig. 1. Kidney function profile in aging. a, Analysis of body weight in female and male, old and young mice (n=5-6). **b,c,** Bar charts showing kidney function profiles, represented as concentrations of creatinine (Cr) (**b**) and BUN (**c**) in sera of female and male, old and young mice (n=5-6). Error bars represent SD (**a-c**). *P* values were calculated using two-way ANOVA followed by Tukey's post *hoc* test (**a-c**). (*P<0.05, ****P<0.0001, ns, not significant). Source data are provided as a Source Data file.

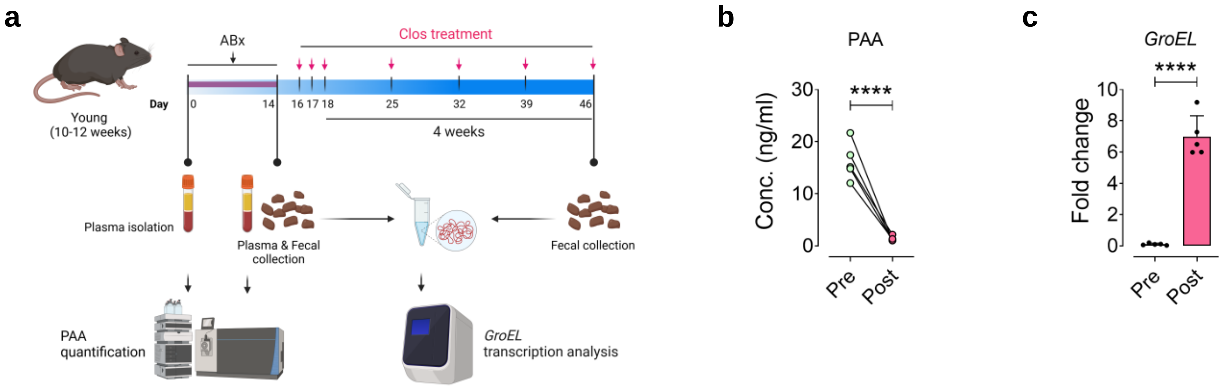

Supplementary Fig. 2. *In vivo* colonization establishes *Clostridium* sp. ASF356 in the intestine of colonized mice. a, Schematic diagram of the experimental setting: 10-12-week-old C57BL/6J mice were pre-treated with antibiotics and then colonized with *Clostridium* sp. ASF356 for 4 weeks. Plasma samples were collected before and after ABx treatment (2 weeks), followed by LC-MS/MS quantification of PAA. Fecal content were also collected at baseline (pre-colonization) and post-colonization (4 weeks after the onset of mono-colonization) from the distal colon to test the enrichment of the bacterium in the intestine of colonized mice. To verify the successful mono-colonization, the expression of the *GroEL* gene, which is unique and highly specific to *Clostridium sp.* ASF356 and encodes the conserved molecular chaperone GroEL, was examined. **b,** Plasma PAA levels significantly decline after 14 days of ABx treatment (n=5). **c,** qPCR analysis reveals a significant increase in *GroEL* expression, indicating the enrichment of *Clostridium* sp. ASF356 in fecal samples 4 weeks post-colonization compared to baseline levels (n=5). Error bars represent SD (**b**). P values were calculated using a two-tailed unpaired Student's t-test (**b,c**). Image created with BioRender.com (**a**). (*$P<0.05$, ****$P<0.0001$*). Source data are provided as a Source Data file.

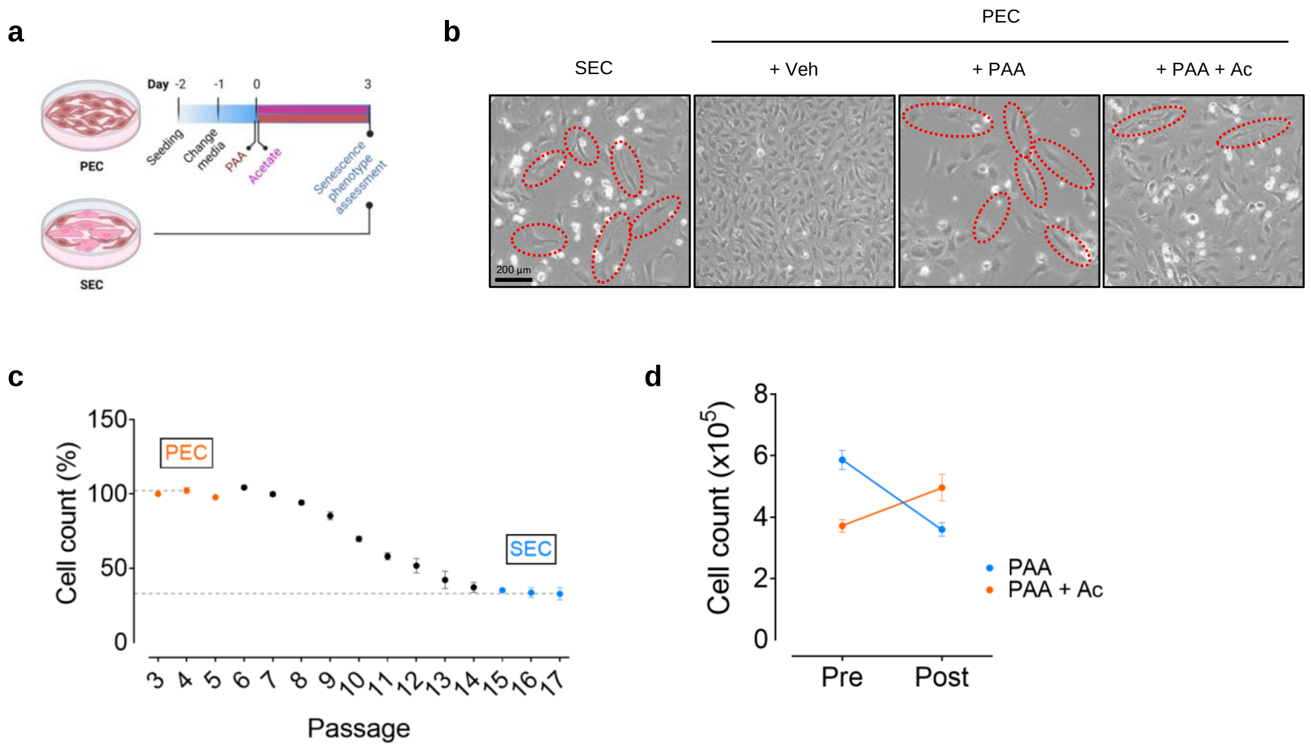

Supplementary Fig. 3. PAA induces senescence phenotype in endothelial cells. a, Schematic diagram of the experimental setting: SECs, PECs, PAA-treated PECs (10 µM, for 72 h), and PAA+Acetate-treated PECs (PAA: 10 µM, sodium acetate: 3 µM, for 72 h) were assessed for senescence phenotype. **b,** Representative bright-field images depicting cellular senescence-like phenotype, including enlarged, flattened, and multinucleated appearance, in PECs treated with PAA at the magnitude seen in replicative SECs. The images demonstrate that sodium acetate reduces the number of cells with morphologically senescence-like phenotype in PAA-exposed PECs (n=6). **c,** HAECs were counted at each passage from p.3 to p.17 and reported as percentage (%). The cells at passages 3 to 5 were characterized as PEC, while those at passages 15 to 17 were considered as SEC (n=6). **d,** PECs were counted at pre- and post-PAA treatment (72 h after the cessation of PAA treatment); PAA-treated PECs were counted before and after exposure to sodium acetate (n=6). Scale bar, 200 µm. Red circles indicate senescent-like cells. Experiments were triplicated independently. Statistical analysis was performed with a one-way ANOVA followed by Tukey's post *hoc* test (**c**) and a two-tailed unpaired Student's *t*-test (**d**). Image created with BioRender.com (**a**). Source data are provided as a Source Data file.

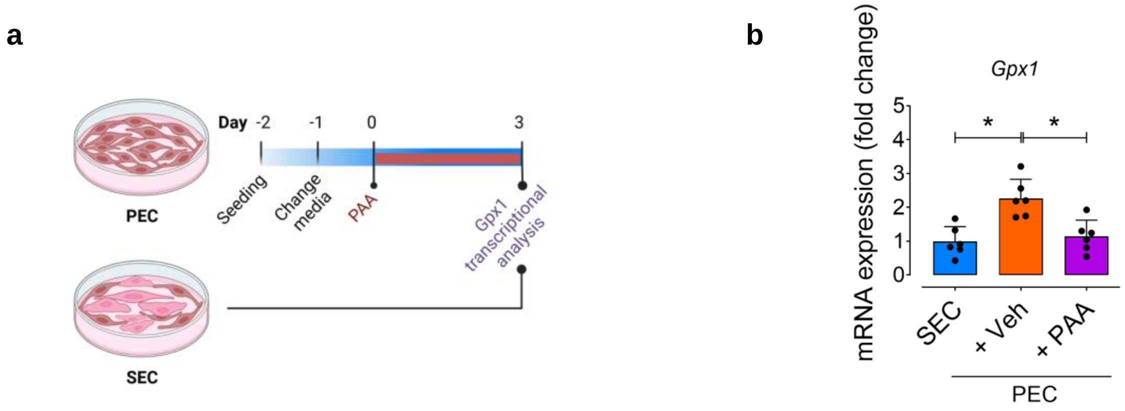

Supplementary Fig. 4. PAA induces oxidative stress by downregulating antioxidant defense in PECs. a, Schematic diagram of the experimental setting: SECs, PECs, and PAA-treated PECs (10 μM, for 72 h) were subjected to *Gpx1* transcriptional analysis. **b,** qPCR analysis demonstrating the downregulation of *Gpx1* in PECs in response to PAA (n=6). Error bars represent SD (**a**). Data represent triplicated biologically independent experiments. *P* values were calculated using one-way ANOVA followed by Tukey's post *hoc* test (**b**). Image created with BioRender.com (**a**). (**P*<0.05). Source data are provided as a Source Data file.

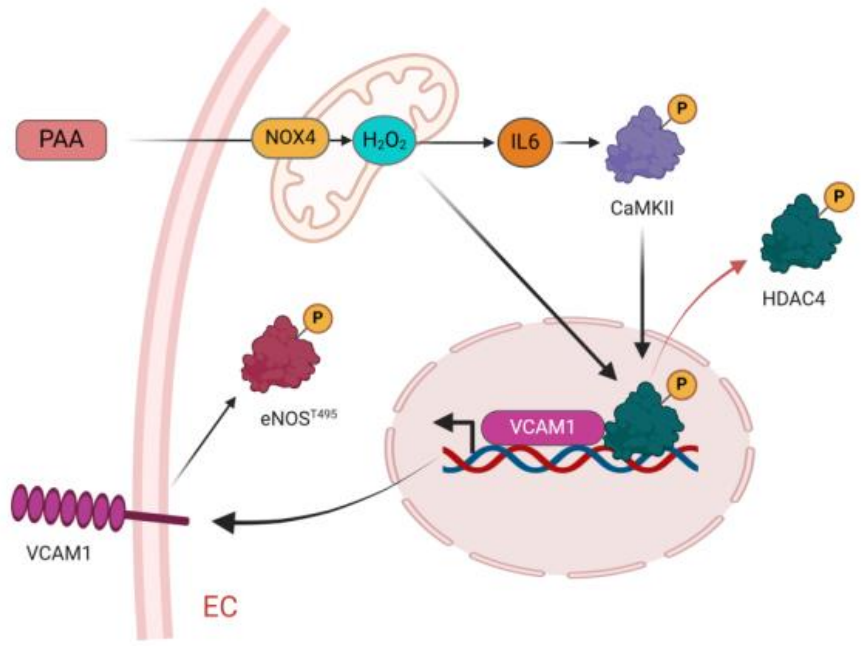

Supplementary Fig. 5. Summary scheme outlining the proposed signaling pathway by which gut-derived PAA induces H_2O_2-mediated IL6 overexpression in proliferating endothelial cells. PAA both directly, by H_2O_2, and indirectly, by IL6, interacts with CaMKII and subsequently stimulates HDAC4 phosphorylation and its nuclear export. It de-represses VCAM1 and reduces phosphorylation of its downstream regulator of endothelial function, eNOS, at Ser1177. Image created with BioRender.com.

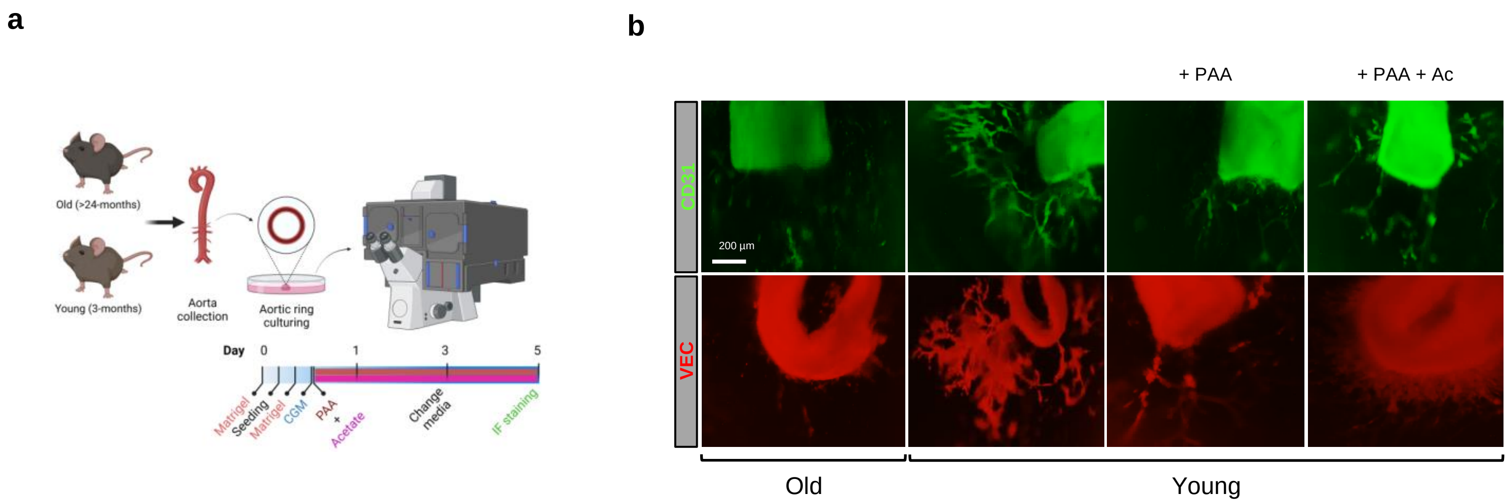

Supplementary Fig. 6. PAA reduces aortic endothelial sprouting. a, Schematic diagram of the experimental setting: aortic rings from old and young mice were subjected to PAA (10 µM) for 72 h and tested for endothelial sprouting, followed by CD31 (green) and VEC (red) immunofluorescence staining. **b,** CD31 and VEC immunofluorescence staining in mouse aortas showing decreased endothelial sprouting after incubation of young aortic rings with PAA as opposed to vehicle. Immunofluorescence images reveal that co-treatment with sodium acetate markedly restores angiogenic capacity of aortic CD31, VEC-positive ECs (n=6). Scale bar, 200 µm. Experiments were triplicated independently. Image created with BioRender.com (**a**).

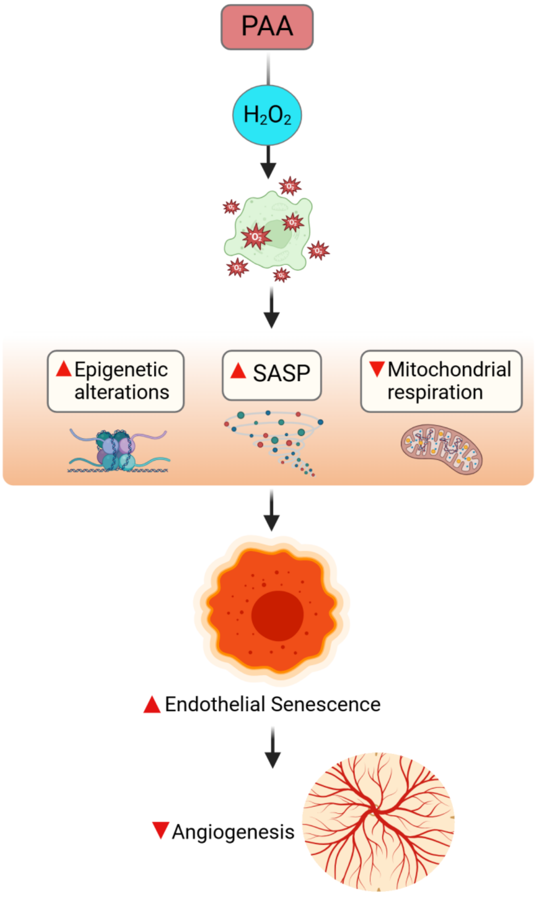

Supplementary Fig. 7. Summary scheme outlines that PAA, through mitochondrial H_2O_2 production, stimulates the SASP-regulated epigenetic responses and impairs mitochondrial respiration, leading to premature endothelial senescence and angiogenic incompetence. Scale bars, 200 μm. Data were determined in 6 micrographs from 3 different plates and represent triplicated biologically independent experiments. Image created with BioRender.com.

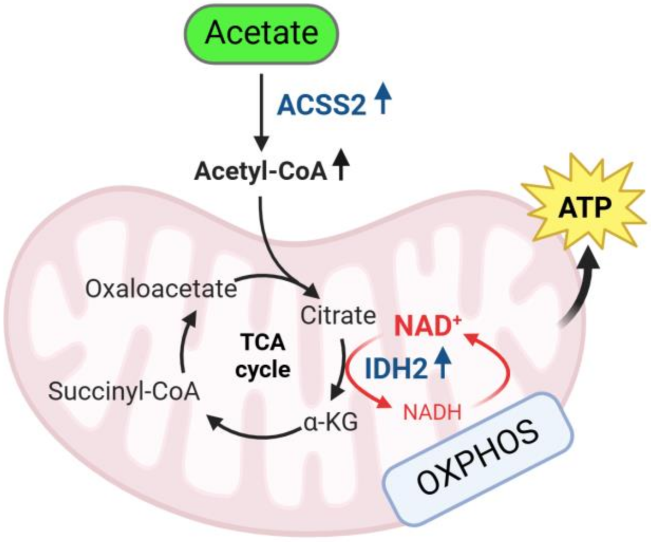

Supplementary Fig. 8. Summary scheme outlines that sodium acetate boosts the TCA cycle by upregulating ACSS2 and acetyl-coA levels that leads to the stimulation of mitochondrial enzymes including IDH2 as part of mitochondrial adaptation. While IDH2 contributes to NAD^+ consumption to generate NADH, the acetate-driven enhancement of oxidative phosphorylation and ATP biosynthesis, as evidenced by enhanced OCR, creates a feedback loop to regenerate NAD^+. Image created with BioRender.com.

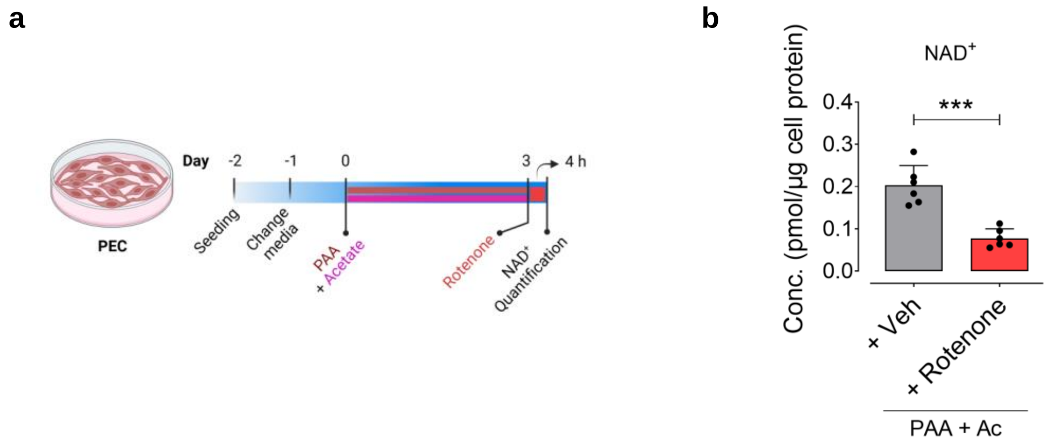

Supplementary Fig. 9. NAD+ recycling by acetate is ETC-dependent. a, Schematic diagram of the experimental setting: PECs were treated with the mitochondrial electron transport chain (ETC) complex I inhibitor, rotenone (1 µM, for 4 h), in the presence of PAA (10 µM, for 72 h) plus sodium acetate (3 µM, for 72 h), followed by relative NAD+ measurement. **b,** Colorimetric assay demonstrates that rotenone significantly reduces NAD+ levels in PECs co-treated with sodium acetate and PAA compared to those exposed to PAA alone (n=6). Error bars represent SD (**b**). Experiments were triplicated independently. *P* values were calculated using a two-tailed unpaired Student's *t*-test (**b**). Image created with BioRender.com (**a**). (****P*<0.001). Source data are provided as a Source Data file.

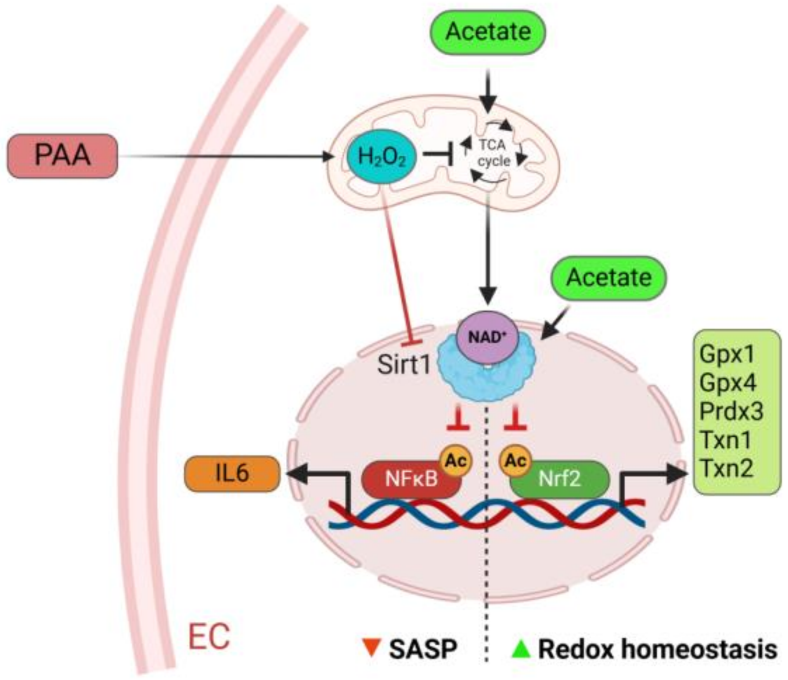

Supplementary Fig. 10. Summary scheme outlines that sodium acetate boosts TCA cycle function and increases NAD^+ bioavailability in mitochondria, activating Sirt1 and biases the system towards deacetylation of downstream targets. Sirt1 facilitates nuclear Nrf2 translocation that recruits transcriptional machinery required for expression of antioxidant target genes, ameliorating PAA-induced mitochondrial oxidative stress (Right). Sodium acetate also exhibits senomorphic effects by Sirt1-mediated deacetylation of NFκB and further downregulation of the SASP component IL6, which was increased in the presence of PAA (Left). Image created with BioRender.com.

Supplementary Table 1. Nucleotide sequence of primers used in this study

Gene-specific forward and reverse primers for RT-PCR amplification. *Gapdh*, Glyceraldehyde 3-phosphate dehydrogenase; *Gpx*, Glutathione peroxidase; *IL1A*, Interleukin 1-alpha; *IL1B*, Interleukin 1-beta; *IL6*, Interleukin 6; *CDKN2A*, p16; *CDKN2D*, p19; *CDKN1A*, p21; *Prdx*, Peroxiredoxin; *TNF*, Tumor necrosis factor-α; *Txn*, Thioredoxin; *VCAM1*, Vascular cell adhesion molecule 1.

Gene	Sequence
Gapdh	AACAGCAACTCCCACTCTTC
	CCTGTTGCTGTAGCCGTATT
Gpx1	CGACATCGAACCTGACATAGAA
	CAGAGTGCAGCCAGTAATCA
Gpx3	TTGATGGGGAGGAGTACATCC
	AGACCGAATGGTGCAAGCTC
Gpx4	ACAAGAACGGCTGCGTGGTGAA
	GCCACACACTTGTGGAGCTAGA
IL1A	GATACAGAGCACCACAGAGAAC
	GGCTAAGGAAACCCTAGGAAAG
IL1B	TCCCAAAGTGCTGGGATTAC
	GAAAGCTCAGAGAGGAGGAAAG
IL6	GCCTGCATTAGGAGGTCTTT
	CCTGACACCAGCAAAGGATAA
p16 (CDKN2A)	GGTTCTCGCAGTACCATTGA
	CTGACTTCTGAGGTGGGTTTAG

Gene	Sequence
p19 (CDKN2D)	GTCTCTCCGTTTCCCTTTCTTC
	GCTGGGCGCTTTATTTCATTC
p21 (CDKN1A)	GGGTGCGGTGATGGATAAA
	ACTGCTGAGAACAGGAAGAAC
Prdx1	CGTCTTGTTCTTGCCTGGTG
	ACATCTTCCTATCAGCAGTCCC
Prdx3	GCA GAT TTC CCG AGA CTA CG
	TAG GAG AAT CCG GTG TCC AG
TNF	CCCTCGATGAAGCCCAATAAA
	CCCTCCCTCCATTCCTTAGATA
Txn1	GCAGCACTGAGTGGTCAAAA
	TGCCTCAATTGCTCTCTCCT
Txn2	GGAGAGAACAGCAGGTAGAAAG
	GTGCCTGTAATCTCAGCTACTC
VCAM1	CCACTTGCCATCCTACTCTATTC
	CAAGAGGGCTTAGGAGATTAAGG

Supplementary Table 2. siRNAs used in this study

**siRNA**
OnTARGETplus smart pool for CaMKII (siCaMKII; Dharmacon, L-004942-00-0005)
OnTARGETplus smart pool for VCAM1 (siVCAM1; Dharmacon, L-013351-00-0005)
OnTARGETplus smart pool for Sirt1 (siSirt1; Dharmacon, L-003540-00-0005)
OnTARGETplus smart pool for RELA proto-oncogene, encoding the NF-kB subunit p65 (siNFκB; Dharmacon, L-003533-00-0005)
OnTARGETplus non-targeting scrambled control siRNA (siNeg; Dharmacon, D-001810-10-05)

Supplementary Table 3. Antibodies and reagents used in this study

Antibodies	Reagents and Chemicals
anti-phospho-CaMKII Thr286 (1:1000; CST, 12716)	ML385 (Sigma-Aldrich, SML1833)
anti-total-CaMKII (1: 1000; CST, 4436)	Phenylacetic acid (Sigma-Aldrich, P16621)
anti-phospho-HDAC4 Ser632 (1:1000; CST, 3424)	Sodium acetate (Sigma-Aldrich, S2889)
anti-total-HDAC4 (1:1000; CST, 7628)	Dasatinib (Sigma-Aldrich, CDS023389)
anti-H3 (1:1000; CST, 9715)	Quercetin (Sigma-Aldrich, Q4951)
anti-phospho-eNOS Ser1177 (1:1000; CST, 9517)	Oligomycin (Sigma-Aldrich, O4876)
anti-phospho-eNOS Thr495 (1:1000; CST, 9574)	FCCP (Sigma-Aldrich, C2920)
anti-total-eNOS (1: 1000; CST, 32027)	Antimycin A (Sigma-Aldrich, A8674)
anti-phospho-Histone H2A.X Ser139 (1:1000; CST, 80312)	Rotenone (Sigma-Aldrich, R8875)
anti-Acetyl-NF-κB p65 (Lys310) (1:1000; CST, 3045)	Acetylcholine chloride (Sigma-Aldrich, A6625)
anti-total-NF-κB p65 (1:1000; CST, 8242)	Human recombinant IL6 (STEMCELL Technologies, 78050)
anti-ACSS2 (1:1000; CST, 3658)	D-alanine (Oakwood Chemical, 005452)
anti-IDH2 (1:1000; CST, 56439)	L-alanine (Oakwood Chemical, 080323)
anti-Sirt1 (1:1000; CST, 9475)	Lipofectamine RNAiMAX transfection reagent (Invitrogen, 13778150)
anti-Nrf2 (1:1000; CST, 12721)	EBM-2 culture medium (Lonza, 00190860)
anti-Col3a1 (1:1000; CST, 30565)	EGM-2 Endothelial Cell Growth Medium-2 BulletKit^{TM} (Lonza, CC-3162/6)
anti-MMP-9 (1:1000; CST, 3852)	Collagenase type II (Sigma-Aldrich, C2-28)
anti-VCAM-1 (1:1000; Invitrogen, MA5-31965)	
anti-IL6 (1:1000; Invitrogen, M-620)	
anti-NADPH oxidase 4 (NOX4) (1:1000; Invitrogen, PA5-72816)	
anti-hTERT (1:500; Invitrogen, MA5-16034)	
anti-VE Cadherin (1:100; Invitrogen, PA5-19612)	
anti-CD31 (1:100; eBioscience, 14-0311-82)	
anti-Histone H3ac (Pan-Acetyl) (1:500; Santa Cruz Biotechnology, sc-518011)	
anti-phospho-HDAC4 Ser632 (1:100; abcam, ab39408-1001)	
anti-p16INK4a (1:100 and 1:1000; abcam, ab211542),	
conjugated secondary anti-Alexa Fluor® 488 (1:2000; abcam, ab150157)	
conjugated secondary anti-Alexa Fluor® 594 (1:2000; abcam, ab150080)	
conjugated secondary anti-Alexa Fluor® 647 (1:2000; abcam, ab150075)	
anti-goat anti-rabbit IgG-HRP (1:2000; Southern Biotechnology, 4030-05)	
anti-goat anti-mouse IgG-HRP (1:2000; Southern Biotechnology, 1036-05)	
