# Supplementary material for: Gut microbiota-dependent increase in phenylacetic acid induces endothelial cell senescence during aging
Source: Nat Aging. 2025 May 12;5(6):1025–45. doi: 10.1038/s43587-025-00864-8 (PMC12176623; doi:10.1038/s43587-025-00864-8)
Supplement: Supplementary file 2 — Reporting Summary [file 43587_2025_864_MOESM2_ESM.pdf]

nature portfolio

Corresponding author(s): Seyed Soheil Saeedi Saravi
Jürg H. Beer

Last updated by author(s): Mar 21, 2025

Reporting Summary

Nature Portfolio wishes to improve the reproducibility of the work that we publish. This form provides structure for consistency and transparency in reporting. For further information on Nature Portfolio policies, see our Editorial Policies and the Editorial Policy Checklist.

Statistics

For all statistical analyses, confirm that the following items are present in the figure legend, table legend, main text, or Methods section.

n/a	Confirmed	
☐	☒	The exact sample size (*n*) for each experimental group/condition, given as a discrete number and unit of measurement
☐	☒	A statement on whether measurements were taken from distinct samples or whether the same sample was measured repeatedly
☐	☒	The statistical test(s) used AND whether they are one- or two-sided<br>*Only common tests should be described solely by name; describe more complex techniques in the Methods section.*
☐	☒	A description of all covariates tested
☐	☒	A description of any assumptions or corrections, such as tests of normality and adjustment for multiple comparisons
☐	☒	A full description of the statistical parameters including central tendency (e.g. means) or other basic estimates (e.g. regression coefficient) AND variation (e.g. standard deviation) or associated estimates of uncertainty (e.g. confidence intervals)
☐	☒	For null hypothesis testing, the test statistic (e.g. *F*, *t*, *r*) with confidence intervals, effect sizes, degrees of freedom and *P* value noted<br>*Give P values as exact values whenever suitable.*
☒	☐	For Bayesian analysis, information on the choice of priors and Markov chain Monte Carlo settings
☒	☐	For hierarchical and complex designs, identification of the appropriate level for tests and full reporting of outcomes
☐	☒	Estimates of effect sizes (e.g. Cohen's *d*, Pearson's *r*), indicating how they were calculated

Our web collection on statistics for biologists contains articles on many of the points above.

Software and code

Policy information about availability of computer code

Data collection	CellRox green imaging: LAS X Office 1.4.6.28433 (Leica);<br>Opal fluorescence imaging: Phenochart 1.2.0 (Akoya Biosciences);<br>HyPer7.2 Fluorescence Imaging: Metafluor Software (Molecular Devices);<br>Ex vivo vasorelaxation assay: LabChart v7.2.5 (AD Instruments, Inc.)
Data analysis	Metabolites Identification: Tracefinder 3.2 (Thermo Scientific);<br>Metagenomic Analysis: Trimmomatic v0.39, https://ftp.ncbi.nlm.nih.gov/genomes/all/GCF/000/001/635/GCF_000001635.27_GRCm39/GCF_000001635.27_GRCm39_genomic.fna.gz;<br>Taxonomic Profiling: kraken2/Bracken2 (https://ezmeta.unige.ch/CMMG/Kraken2db/cmmg), MetaPhlAn v.4.0 & Bowtie 2 v.2.3.4.3 pipelines;<br>KEGG Functional annotations: https://ezmeta.unige.ch/CMMG/functional_annotations/mouse/annotations.tsv;<br>Vegan v2.5-5 package (https://Github.com/vegan);<br>Fecal Acetate Level Analysis: EZChrom A.04.10 (Agilent);<br>GraphPad Prism 9 (v.9.5.1, La Jolla, CA, USA);<br>R v1.26.1 (Vienna, Austria, 2017);<br>R package 'lmerTest' (v3.1.3, function 'lmer');<br>ImageJ software 1.54j (Fiji).

For manuscripts utilizing custom algorithms or software that are central to the research but not yet described in published literature, software must be made available to editors and reviewers. We strongly encourage code deposition in a community repository (e.g. GitHub). See the Nature Portfolio guidelines for submitting code & software for further information.

Data

Policy information about availability of data

All manuscripts must include a data availability statement. This statement should provide the following information, where applicable:

- Accession codes, unique identifiers, or web links for publicly available datasets
- A description of any restrictions on data availability
- For clinical datasets or third party data, please ensure that the statement adheres to our policy

We declare that Source Data and Supplementary Information are provided with this paper. All data from shotgun metagenomics analyses in this study have been deposited in the National Center for Biotechnology Information Sequence Read Archive (SRA) under accession number PRJNA1242241. Screening of K00169 and K00179 was conducted using KEGG annotations, which are accessible at https://ezmeta.unige.ch/CMMG/functional_annotations/mouse/annotations.tsv. The targeted metabolomics data, including Thermo's raw data files and processed datasets analyzed by Tracefinder 3.2, have been securely stored on an institutional research repository at University of Lausanne. These data are available upon request from the corresponding authors and subject to institutional data-sharing policies. The human study data used in this research are maintained by the Department of Twin Research at King's College London and are not publicly available due to patient privacy and data protection regulations. Researchers seeking access must apply through the established procedures outlined by the Wellcome Trust, following the guidelines provided at https://twinsuk.ac.uk/resources-for-researchers/access-our-data/. Access is granted upon approval and may require specific ethical and governance clearances. All other data and reagents that support the findings of this study are available upon reasonable request from the corresponding authors.

Research involving human participants, their data, or biological material

Policy information about studies with human participants or human data. See also policy information about sex, gender (identity/presentation), and sexual orientation and race, ethnicity and racism.

Reporting on sex and gender	The human analyses were performed on both female and male subjects.
Reporting on race, ethnicity, or other socially relevant groupings	The reports are provided by the the Department of Twin Research at King's College London.
Population characteristics	The TwinsUK cohort includes more than 14,000 adult twins of the same sex between 18 and 95 years old. All participants received no compensation and provided written informed consent.
Recruitment	Data were collected by the TwinsUK biobank. Participants were recruited from the general UK population through national media campaigns. Other than the subjects being mostly females, no selection biases can be expected to impact results.
Ethics oversight	The study was approved by the NRES Committee London-Westminster (REC Reference No.: EC04/015). All participants in TwinsUK Cohort received no compensation and provided written informed consent.

Note that full information on the approval of the study protocol must also be provided in the manuscript.

Field-specific reporting

Please select the one below that is the best fit for your research. If you are not sure, read the appropriate sections before making your selection.

☒ Life sciences ☐ Behavioural & social sciences ☐ Ecological, evolutionary & environmental sciences

For a reference copy of the document with all sections, see nature.com/documents/nr-reporting-summary-flat.pdf

Life sciences study design

All studies must disclose on these points even when the disclosure is negative.

Sample size	No statistical methods were used to pre-determine sample sizes, but similar sample sizes were used in previous publications (Saeedi Saravi, S.S. et al., 2023, 2021; Shabanian, K. et al., 2024). In TwinsUK human study, participants that exhibited values below the detection level (0) for PAA or PAGln were considered as not available. Accordingly, PAA and PAGln were measured in plasma samples of 7,303 subjects by Metabolon using a non-targeted UPLC–MS/MS platform.
Data exclusions	No animals or data points were excluded from experiments or analyses.
Replication	All attempts at replication were successful. At least three independent triplicated experiments were performed for each experimental set-up.
Randomization	No method of randomization was used to assign animals to experimental groups.
Blinding	The investigators in this study were blinded to the conditions of the experiments, except for oral gavage (for Clos, PAA, or D+Q administration) to avoid cross-contamination within the groups.

Reporting for specific materials, systems and methods

We require information from authors about some types of materials, experimental systems and methods used in many studies. Here, indicate whether each material, system or method listed is relevant to your study. If you are not sure if a list item applies to your research, read the appropriate section before selecting a response.

Materials & experimental systems

n/a	Involved in the study
☐	☒ Antibodies
☐	☒ Eukaryotic cell lines
☒	☐ Palaeontology and archaeology
☐	☒ Animals and other organisms
☒	☐ Clinical data
☒	☐ Dual use research of concern
☒	☐ Plants

Methods

n/a	Involved in the study
☒	☐ ChIP-seq
☒	☐ Flow cytometry
☒	☐ MRI-based neuroimaging

Antibodies

Antibodies used

anti-phospho-CaMKII Thr286 (1:1000; CST, 12716)
anti-total-CaMKII (1: 1000; CST, 4436)
anti-phospho-HDAC4 Ser632 (1:1000; CST, 3424)
anti-total-HDAC4 (1:1000; CST, 7628)
anti-H3 (1:1000; CST, 9715)
anti-phospho-eNOS Ser1177 (1:1000; CST, 9517)
anti-phospho-eNOS Thr495 (1:1000; CST, 9574)
anti-total-eNOS (1: 1000; CST, 32027)
anti-phospho-Histone H2A.X Ser139 (1:1000; CST, 80312)
anti-Acetyl-NF-κB p65 (Lys310) (1:1000; CST, 3045)
anti-total-NF-κB p65 (1:1000; CST, 8242)
anti-ACSS2 (1:1000; CST, 3658)
anti-IDH2 (1:1000; CST, 56439)
anti-Sirt1 (1:1000; CST, 9475)
anti-Nrf2 (1:1000; CST, 12721)
anti-Col3a1 (1:1000; CST, 30565)
anti-MMP-9 (1:1000; CST, 3852)
anti-VCAM-1 (1:1000; Invitrogen, MA5-31965)
anti-IL6 (1:1000; Invitrogen, M-620)
anti-NADPH oxidase 4 (NOX4) (1:1000; Invitrogen, PA5-72816)
anti-hTERT (1:500; Invitrogen, MA5-16034)
anti-VE Cadherin (1:100; Invitrogen, PA5-19612)
anti-CD31 (1:100; eBioscience, 14-0311-82)
anti-Histone H3ac (Pan-Acetyl) (1:500; Santa Cruz Biotechnology, sc-518011)
anti-phospho-HDAC4 Ser632 (1:100; abcam, ab39408-1001)
anti-p16INK4a (1:100 and 1:1000; abcam, ab211542),
conjugated secondary anti-Alexa Fluor® 488 (1:2000; abcam, ab150157)
conjugated secondary anti-Alexa Fluor® 594 (1:2000; abcam, ab150080)
conjugated secondary anti-Alexa Fluor® 647 (1:2000; abcam, ab150075)
anti-goat anti-rabbit IgG-HRP (1:2000; Southern Biotechnology, 4030-05)
anti-goat anti-mouse IgG-HRP (1:2000; Southern Biotechnology, 1036-05)

Validation

According to the data sheet, all the antibodies used for immunoblotting, immunofluorescence staining, and Opal Multiplex immunofluorescence can detect target
proteins. Also, Alexa Fluor antibodies are suitable for tissue immunostaining according to the data sheet.
All primary and secondary antibodies were validated for the application (see citations provided on manufactures websites for the above outlined catalogue numbers).

Eukaryotic cell lines

Policy information about cell lines and Sex and Gender in Research

Cell line source(s)

Primary human aortic endothelial cells (HAEC; Lonza, CC-2535)

Authentication

Primary human aortic endothelial cells (HAEC; Lonza, CC-2535)

Mycoplasma contamination

Cultured cells were regula?y tested for mycoplasma contamination. We have confirmed the negative results for the cells used in our experiments.

Commonly misidentified lines (See ICLAC register)	N/A

Animals and other research organisms

Policy information about studies involving animals; ARRIVE guidelines recommended for reporting animal research, and Sex and Gender in Research

Laboratory animals	We have reported necessary information appropriately in the manuscript. 8 week old wild-type C57BL/6J mice were obtained from the Jackson Laboratory and maintained in our facility for specific times for normal aging modeling. All mice were individually housed in controlled environments in plexiglass cages under a strict 12 h:12 h light/dark cycles at an ambient temperature of 23 ± 1°C and humidity of 55 ± 10%, and fed standard chow diet (Research Diets) until 3 (as Young) or >24 (as Old) months of age. Mice had access to drinking water and food ad libitum.<br>10-12-week-old wild-type C57BL/6J mice were obtained from the Jackson Laboratory and maintained in our facility for in vivo bacterial colonization.
Wild animals	There was no wild animal used in the study.
Reporting on sex	Equal numbers of both female and male mice were used.
Field-collected samples	There were no field-collected samples used in the study.
Ethics oversight	Our research complies with all ethical regulations approved by the Institutional Animal Care and Use Committee at University of Zurich and the Cantonal Ethics Commission Zurich (ZH023/2023 and ZH241/19).

Note that full information on the approval of the study protocol must also be provided in the manuscript.

Plants

Seed stocks	*Report on the source of all seed stocks or other plant material used. If applicable, state the seed stock centre and catalogue number. If plant specimens were collected from the field, describe the collection location, date and sampling procedures.*
Novel plant genotypes	*Describe the methods by which all novel plant genotypes were produced. This includes those generated by transgenic approaches, gene editing, chemical/radiation-based mutagenesis and hybridization. For transgenic lines, describe the transformation method, the number of independent lines analyzed and the generation upon which experiments were performed. For gene-edited lines, describe the editor used, the endogenous sequence targeted for editing, the targeting guide RNA sequence (if applicable) and how the editor was applied.*
Authentication	*Describe any authentication procedures for each seed stock used or novel genotype generated. Describe any experiments used to assess the effect of a mutation and, where applicable, how potential secondary effects (e.g. second site T-DNA insertions, mosiacism, off-target gene editing) were examined.*
